# The absence of (TCAGGG)_n_ repeats in some telomeres, combined with variable responses to NR2F2 depletion, suggest that this nuclear receptor plays an indirect role in the alternative lengthening of telomeres

**DOI:** 10.1038/s41598-020-77606-w

**Published:** 2020-11-26

**Authors:** Ahmed S. N. Alhendi, Nicola J. Royle

**Affiliations:** Department of Genetics and Genome Biology, University of Leicester, Leicester, LE1 3HE UK

**Keywords:** Cancer, Cell biology, Genetics, Molecular biology

## Abstract

The alternative lengthening of telomeres (ALT) facilitates telomere lengthening by a DNA strand invasion and copying mechanism. The nuclear receptors (NRs), NR2F2 and NR2C2, can bind to (TCAGGG)_n_ variant repeats within telomeres and it has been proposed that this facilitates telomere interactions in ALT+ cells. Here we show that the frequency of cells with detectable NR2F2 and NR2C2 nuclear foci varies considerably between ALT+ cell lines and does not correlate with the level of protein expression. In addition, four of five ALT+ cell lines lack (TCAGGG)_n_ repeats in some telomeres, indicating that direct NR binding does not play a role in ALT at these telomeres. NR2F2-depletion altered the abundance of C-circles and APBs but the direction of the response was inconsistent between three ALT+ cell lines. Moreover, transcriptome analysis following NR2F2-depletion in the ALT+ cell lines revealed different very responses. For example, NR2F2-depletion down-regulated many genes in U2OS cells, consistent with the cell cycle arrest and changes to ALT markers, but these features were not shared by the other two ALT+ cell lines. Among 86 ALT-associated genes, only *MND1* showed consistent down-regulation across three NR2F2-depleted ALT+ cell lines. Altogether our data suggest that NR2F2 does not play a direct role in ALT and we speculate about an alternative role for this NR in a DNA damage response at telomeres.

## Introduction

Telomeres are the protective DNA–protein complexes that cap the ends of linear chromosomes. In human cells the double-stranded DNA component can be subdivided into a proximal region (closest to the centromere) comprising variant or degenerate repeats interspersed with the canonical telomeric repeat (TTAGGG)^1–3^ and the distal region that is assumed to be predominantly (TTAGGG)_n_. Telomeric DNA is terminated by a G-rich single strand overhang, which is required for t-loop formation and capping^4,5^. Degenerate or variant repeats that maintain the 6-base pair periodicity and contain three consecutive G-residues tend to be more abundant in telomeric DNA than others^6^. Repeats such as (TGAGGG), (TCAGGG) and (TTGGGG) often have complex interspersion patterns with (TTAGGG) repeats and these patterns vary between alleles at each chromosome end^2,3,7^. Furthermore short arrays of (CTAGGG)_n_, cause localised, replication-dependent instability in telomeres^8^. Variant repeats are unlikely to bind to TRF1 or TRF2 in vivo, although some may bind to POT1^8^, rendering these proximal regions of telomeres only partially functional in normal cells. In summary, the proximal telomere that includes variant repeats, varies in length and composition between alleles, chromosomes and individuals.

In the absence of an active telomere maintenance mechanism (TMM), telomeres shorten during each cell division until the Hayflick limit (also known as M1) is reached when short telomeres signal DNA damage and the cell enters senescence^9^. Bypass of the M1 checkpoint, through disruption of *TP53* and *RB1* expression, extends the replicative lifespan until the cell reaches crisis (also known as M2). Telomere: telomere fusions that form at crisis^10,11^, contribute to a high level of genome instability and cell death^12^. To survive crisis a TMM must be activated, either telomerase or the Alternate Lengthening of Telomeres (ALT).

The ALT mechanism is utilised by 10–15% of cancers, in particular some sarcomas and tumours of the central nervous system^13,14^. There are a variety of biomarkers that can be used to determine if a cell line or tumour shows ALT activity. These include: highly heterogenous telomere length; the presence of ALT-associated PML bodies (APBs)^15^; high frequency of recombination at telomeres^16,17^ often described as telomere sister-chromatid exchanges^18,19^; extensive presence of Telomere Induced DNA damage Foci (TIFs) that persist through metaphase and cell division^20^; presence of both G-and C-overhangs^21^; altered chromatin modifications in subtelomeric regions associated with increased expression of telomeric repeat containing RNA (TERRA)^22–25^; and a high level of extrachromosomal linear and circular telomeric DNA, in particular C-circles. C-circles are variable sized, partially double-stranded molecules that arise from DNA damage-induced replication fork collapse, which results in sudden cleavage and circularisation during lagging strand synthesis of telomeres in ALT+ cells^21,26,27^.

ALT is a homology directed repair (HDR) mechanism that shows strongest resemblance to Break-Induced Replication (BIR)^28,29^. It involves strand invasion of one telomere that will be lengthened (recipient) into another telomere or sister-telomere (donor), which is used as a template for copying and extension of the invading telomere. As a result of this process, variant repeats, normally located in proximal regions, can become distributed throughout the full length of telomeres and between telomeres^6,17,30–32^. A variety of genes are required for telomere length maintenance in ALT including but not limited to the MRN complex (*MRE11/RAD50/NBS1)*^33^; *POLD3*^34^; BTR (*BLM/TOP3A/RMI1/2*) and its interaction with *FANCM*^35–37^. ALT also requires regulation and localisation TRF1 and TRF2 proteins to APBs via sumoylation by the SMC5/6 complex^38^. Frequent inactivation or loss of *ATRX,* or its partner *DAXX*^39,40^, along with histone H3.3 mutations, demonstrate that telomeric chromatin organisation and correct histone deposition play significant roles in suppressing ALT^41,42^.

Recent studies have identified a variety of routes that are thought to bring telomeres together to facilitate strand invasion during ALT. These include (1) recruitment of RCF-PCNA-POLD3, as expected for BIR^43^; (2) a slower long-range homology searching process that requires RAD51 single-strand filament formation and the HOP1-MND1 heterodimer^44^ and (3) orphan nuclear receptors (NRs) binding to variant (TCAGGG)_n_ repeats within telomeres, facilitating recruitment of ZNF827, a zinc-finger DNA binding protein and the NuRD, nucleosome remodelling and deacetylase protein complex^31,45^.

NRs are a large family of diverse transcriptional activator /repressors that bind to DNA in a sequence specific manner and play many roles in cell regulation, differentiation and organogenesis. The class of NRs that can bind to (TCAGGG) repeats include NR2F2 (COUP-TFII) and NR2C2 (TR4). NR2F2 in particular is known to play significant roles in tumorigenesis through its impact on angiogenesis and the tumour microenvironment^46,47^. Here we show that multiple telomeres appear to lack binding sites for NR2F2 and NR2C2 in four of five ALT+ cell lines. This is consistent with a lower frequency of NR2F2 and NR2C2 foci at telomeres in the nuclei of four out of five ALT+ cell lines. We propose that telomeres lacking NR2F2 and NR2C2 binding sites are unlikely to engage in direct recruitment of ZNF827, the NuRD complex, or other NR2F2 interacting proteins, such as FANCD2^48^. siRNA mediated NR2F2 down-regulation in three ALT+ cell lines had a modest to severe effect on the cell cycle but the effect on various ALT markers was highly inconsistent between the cell lines. Transcriptome analysis showed that NR2F2 depletion had a dramatic effect on gene expression in U2OS cells that was consistent with a reduction in cells in S-phase and near cell cycle arrest. Notably only one ALT-related gene, *MND1*, was down-regulated upon NR2F2 depletion in all three cells lines, possibly suggesting an indirect role for NR2F2 in the regulation of *MND1*. In summary, NR2F2 has a known role in cell cycle regulation that our data corroborate, but its role in the ALT mechanism appears to be more indirect than has been proposed previously. Nevertheless NR2F2 and NR2C2 do bind to some telomeres where they recruit other proteins^31,48^ and we speculate that this may serve a different function.

## Results

### NR2F2 and NR2C2 expression and telomeric localisation in ALT+ cell lines

**Figure 1:**
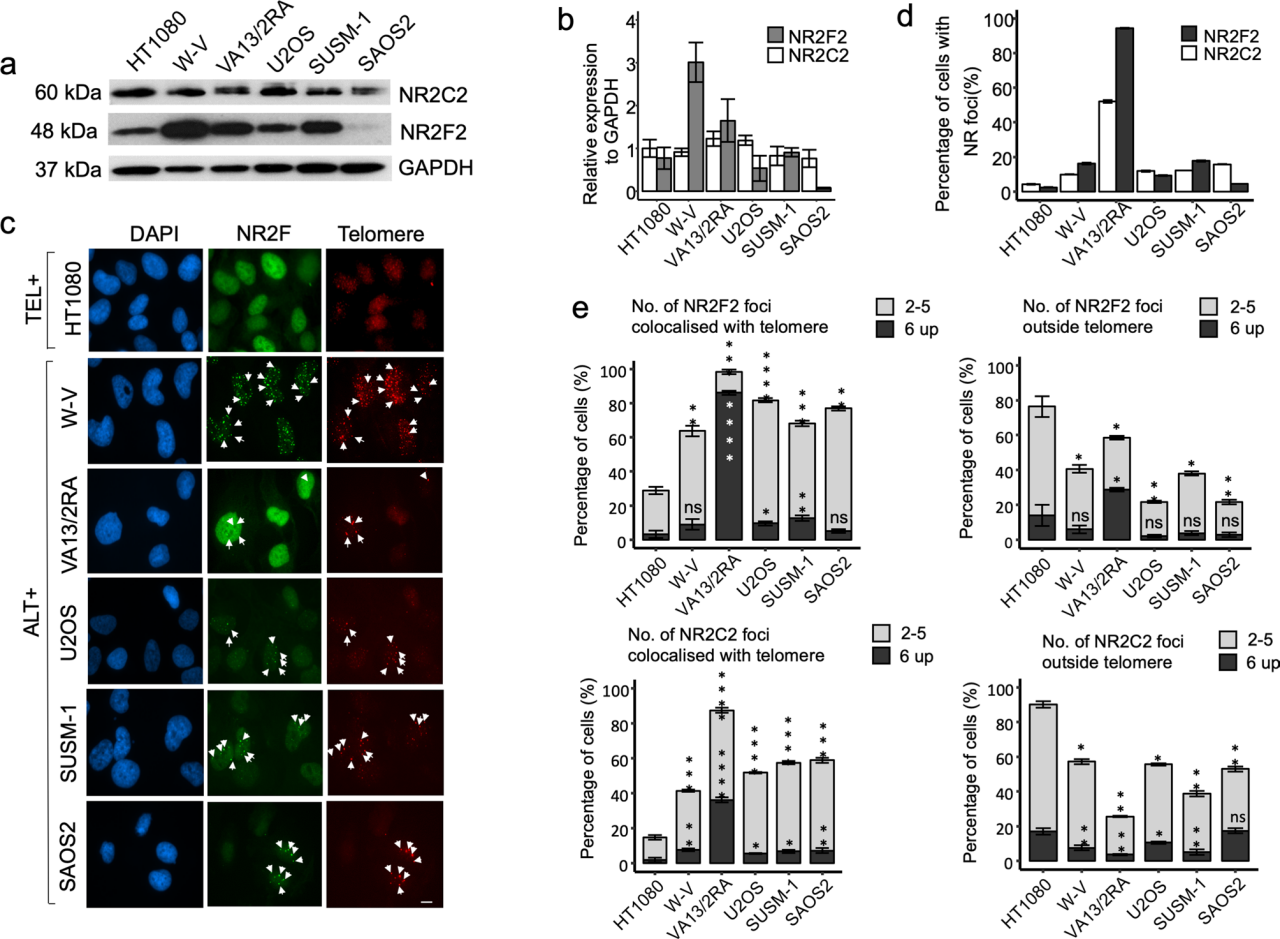
Assessment of NR2C2 and NR2F2 protein expression and localisation at telomeres in one telomerase + (HT1080) and five ALT+ cell lines. (**a**) Western blot detection of NR2F2 and NR2C2 in the cells lines and (**b**) graph showing the quantification of NR2C2 and NR2F2 expression relative to GAPDH (control), N = 3 independent biological replicates, error bars show standard deviation (SD). (**c**) Immunofluorescence detection of NR2F2 foci (green) and telomere foci by Telo-PNA hybridisation (red) in DAPI stained nuclei (blue) from the HT1080 (Tel +) and the VA132RA, W-V, U2OS SUSM-1 and SAOS2 (ALT +) cell lines. Scale bar 10 µm. (**d**) Graph showing the percentage of nuclei with NR2F2 or NR2C2 foci for each cell line. The graph shows the mean ± SD from three independent experiments. (**e**) Cells with NR2F2 or NR2C2 foci (as in d) were then assessed for the location of the foci. The proportion of cells with 2–5 (light grey) or 6 and over foci (dark grey) that did or did not colocalise with telomeric signals are shown for each cell line. Comparative analysis the between the distribution of the NR2F2/NR2C2 foci on and off telomeres was conducted by one-way ANOVA with HT1080 (telomerase +) as the reference group, followed by pairwise t tests between HT1080 and each ALT+ cell line (*P* values = **** < 0.00005; *** < 0.0005; ** < 0.005;* < 0.05; ns > 0.05).

To explore the role of NR2F2 and NR2C2 in ALT+ cell lines, the relative protein expression was measured by western blot analysis in the W-V, WI38VA13/2RA (hereafter abbreviated to VA13/2RA), U2OS, SUSM-1 and SAOS2 ALT+ cell lines and in HT1080 (telomerase +). This showed a fairly consistent NR2C2 expression but considerable variation in NR2F2 expression between cell lines, from barely detectable in SAOS-2 to highly expressed in W-V (Fig. 1a,b). There was no obvious relationship between the NR2F2 and NR2C2 protein expression level and the percentage of nuclei with distinct foci for either NR among the cell lines (Fig. 1c,d, Supplementary Figure S1, Supplementary Figure S2 and Supplementary Table 1). Notably only the VA13/2RA ALT+ cell line showed a high percentage of nuclei with NR foci (NR2F2 94% ± 0.25%; NR2C2 52% ± 0.91%), as reported previously^6,30^. The other ALT+ cell lines showed much lower percentages of cells with NR2F2 or NR2C2 foci, some approaching the low level observed in the HT1080 telomerase + cell line. Distribution analysis was conducted on the nuclei with NR2F2 or NR2C2 foci to investigate colocalisation with telomeres. This demonstrated that, when present, NR2F2 foci colocalised with telomeres in all the cell lines, although ALT+ cell lines (in particular VA13/2RA^6,30^) showed significantly higher proportion of NR2F2 foci at telomeres compared to HT1080. In addition, all cell lines also had foci that did not colocalise with telomeres. Similar trends were observed in nuclei with NR2C2 foci again with VA13/2RA showing the highest proportion of cells with foci at telomeres (Fig. 1d,e, Supplementary Table 1, Supplementary figures 1 and 2).

**Table 1:** Percentage of amplified telomere molecules that contain C-type (TCAGGG) or G-type (TGAGGG) repeats.

Cell line	Total molecules detected	% telomere molecules with C-type repeats (No. C-type + /total)	% telomere molecules with G-type repeats (No. G-type + /total)
**XpYp telomere**			
HT1080	124	0	87 (108/124)
VA13/2RA	254	76 (192/254)	37 (93/254)
W-V	237	47 (111/237)	57 (134/237)
U2OS	192	0	0
SUSM-1	189	0	67 (126/189)
SAOS2	240	0	53 (128/240)
**12q telomere**			
HT1080	150	79 (119/150)	72 (108/150)
VA13/2RA	82	80 (66/82)	0
W-V	115	33 (38/115)	8 (9/115)
U2OS	205	40 (83/205)	51 (105/205)
SUSM-1	198	39 (78/198)	40 (79/198)
SAOS2	85	34 (29/85)	0
**17p telomere**			
HT1080	77	0	0
VA13/2RA	125	90 (113/125)	0
W-V	259	0	0
U2OS	95	74 (70/95)	0
SUSM-1	169	0	13 (22/169)
SAOS2	0	0	0

**Figure 2:**
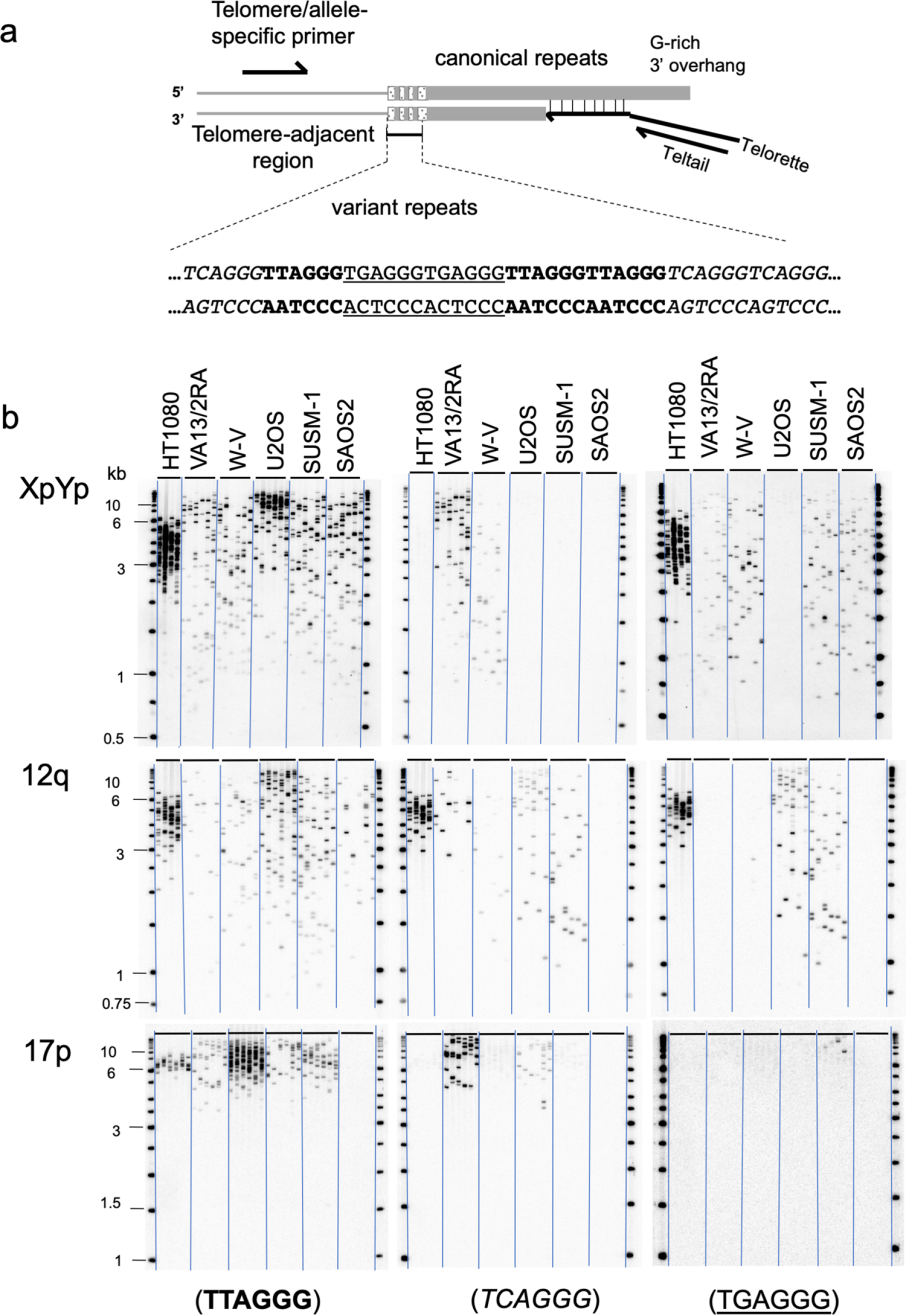
Detection of the variant-repeats, (TCAGGG)_n_ and (TGAGGG)_n_, in molecules amplified from the XpYp, 12q and17p telomeres. (**a**) Diagram showing amplification of telomere molecules by STELA and the location of variant-repeats in the proximal regions of telomeric DNA. (TTAGGG) in bold, (TCAGGG)_n_ in italics and (TGAGGG)_n_ underlined. (**b**) shows amplification of molecules from XpYp, 12q and 17p telomeres using STELA and three identical STELA Southern blots hybridised to ^32^P labelled synthetic probes, comprising (TTAGGG)_n_, (TCAGGG)_n_ or (TGAGGG)_n_ repeats, shows that some but not all telomeres contain potential binding sites for NR2F2 and NR2C2. The absence of STELA products with the 17p6 primer in the SAOS2 cell line, is an example of the polymorphic nature of the primer annealing site. It is likely that the original donor of the SAOS2 cell line had two copies of 17p that both lacked the primer annealing site or alternative the donor had one copy of 17p without and one with a primer annealing site, but the latter was lost during the osteosarcoma development.

### Assessment of telomeres that contain binding sites for NR2F2 and NR2C2.

Given the variable expression and localisation of the two NRs at telomeres in the ALT+ cell lines, the sequence content of XpYp, 12q and 17p telomeres was assessed by specific amplification using Single Telomere Length Analysis (STELA) and hybridisation to DNA probes that detect the canonical (TTAGGG) or the variant repeats (TCAGGG) and (TGAGGG). Only the VA13/2RA cell line showed the presence of (TCAGGG)_n_ repeats in a high percentage of the amplified telomere molecules (Fig. 2, Table 1). The other cell lines all lacked (TCAGGG)_n_ repeats from one or more telomeres. The detection of (TCAGGG)_n_ repeats in approximately 50% of amplified molecules from some telomeres, for example XpYp in W-V, suggested this arose from allelic differences in the progenitor cell line^2^. This was confirmed using allele specific XpYp STELA primers in W-V cell line (Supplementary Figure S3).

### Depletion of NR2F2 has different effects in three ALT+ cell lines

**Figure 3:**
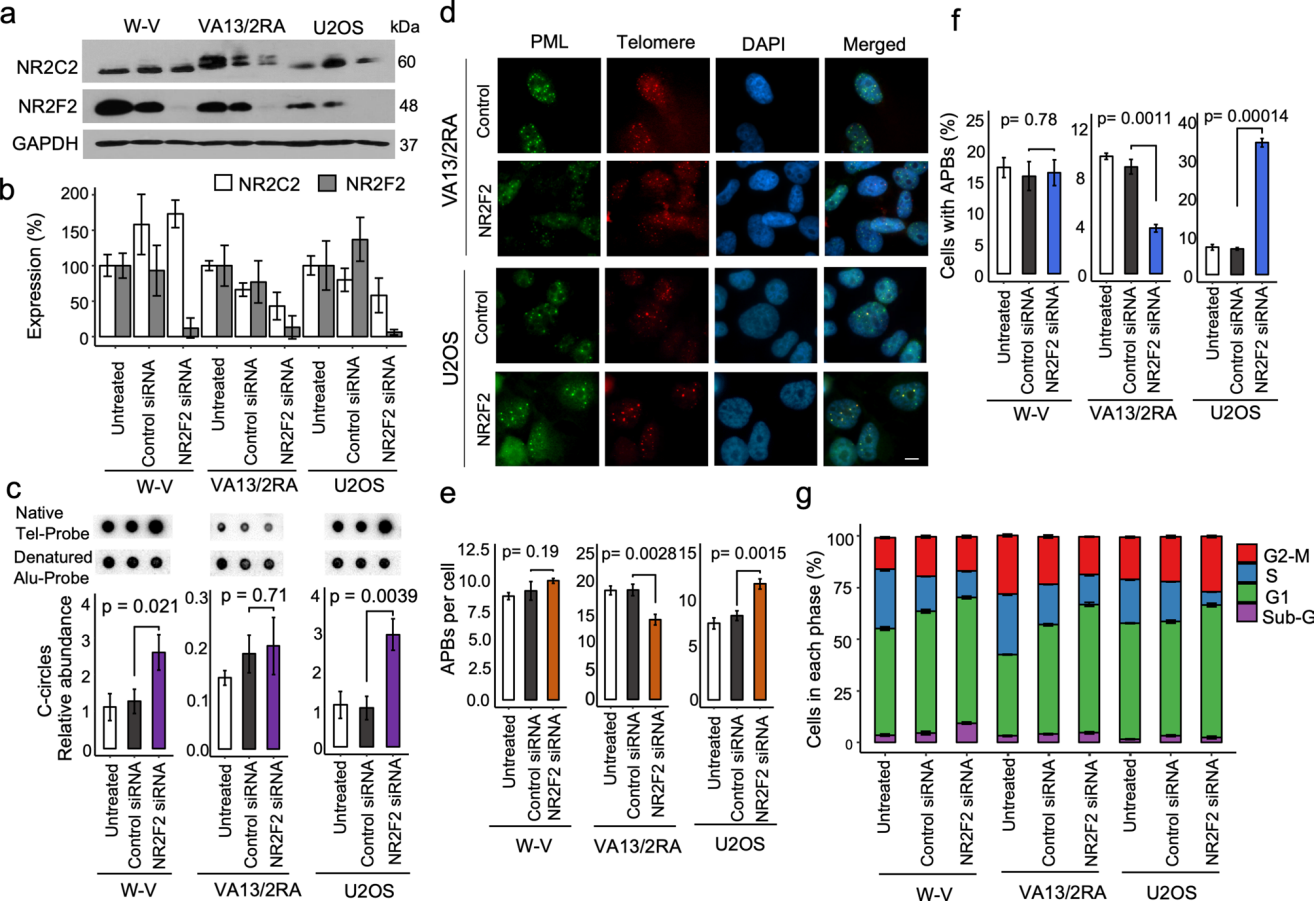
Assessment of ALT markers following transient depletion of NR2F2. (**a**) Western blots showing NR2F2 and NR2C2 protein expression following 72 h treatment with siNR2F2 compared to control or untreated cells. (**b**) Quantification of NR2F2 and NR2C2 levels derived from three biological replicate experiments. (**c**) Example of C-circle dot-blot assays and quantification of the normalised C-circle abundance, mean ± standard deviation from three independent siNR2F2 experiments. (**d**) Examples of APBs detected in control or NR2F2 downregulated in VA13/2RA and U2OS cells. IF staining of the PML (green, Alexa 488) with detection of telomeric DNA using a Telo-PNA probe (Cy3–(CCCTAA)3, red) and counterstained with DAPI (blue). (**e**, **f**) Quantification of APBs upon NR2F2 depletion in the ALT+ cell lines from three independent replicates for siRNA control and siNR2F2 treated and two replicates for the untreated cells. The number of APBs per nucleus is shown as the mean ± SD, and the frequencies of nuclei with APBs shown as a percentage ± SD. (**g**) Cell cycle analysis of the treated and untreated ALT+ cell lines. Graph shows the mean ± standard deviation, from three independent cell cycle analyses.

To investigate the role of NR2F2 in the ALT mechanism, it was depleted from the W-V, VA13/2RA and U2OS ALT+ cell lines by transfection of a siRNA that targets NR2F2 exon 2. To avoid off-target effects, the siNR2F2 was carefully designed to target all known NR2F2 alternative transcripts, with no significant similarities to other genes and five mismatches to the equivalent region of NR1F1, the closest homologous gene (Supplementary Figure S4). Following transfection with the NR2F2 or control siRNA, cells were harvested after 72 h. The siRNA treatment reduced NR2F2 expression in W-V by 89% (± 19.7%), in VA13/2RA by 87% (± 15.1%) and in U2OS by 94% (± 2.3%) but was not accompanied by a significant change in NR2C2 expression across the cell lines (Fig. 3a,b, Supplementary Figure S5). C-circle abundance showed no significant change in VA13/2RA cells following NR2F2 depletion, as reported previously^6^. In contrast, C-circles were significantly increased in the W-V and U2OS NR2F2 depleted cells (Fig. 3c). Assessment of APBs in the three cell lines also showed very different responses to NR2F2 depletion. The percentage of cells with APBs and the number of APBs per cell was unchanged in the treated W-V cells, but both measures were significantly reduced in VA13/2RA (as reported previously^6^) and in contrast, both measures were significantly increased in the treated U2OS cells (Fig. 3 and Supplementary Figure S6).

NR2F2 influences cell proliferation and the cell cycle by binding to the promoter of E2F1, a major regulator of the G1 /S transition, and stimulating its expression^49^. So next we measured changes to the cell cycle by FACs analysis. All three NR2F2 depleted cell lines showed some changes to the cell cycle. Most notable were an increase in dead, sub-G1 cells (from 4.5% to 9.3%, *P* = 0.00173) in the siNR2F2 treated W-V cells and a highly significant decrease in cells in S-phase in siNR2F2 treated U2OS cells (from 19.5% to 6.6%, *P* = 9.0e−07) that was accompanied by increases in G1 and G2-M, suggesting a cell cycle arrest (Supplementary Figure S6).

### Transcriptome analysis in the NR2F2 depleted ALT+ cell lines

**Figure 4:**
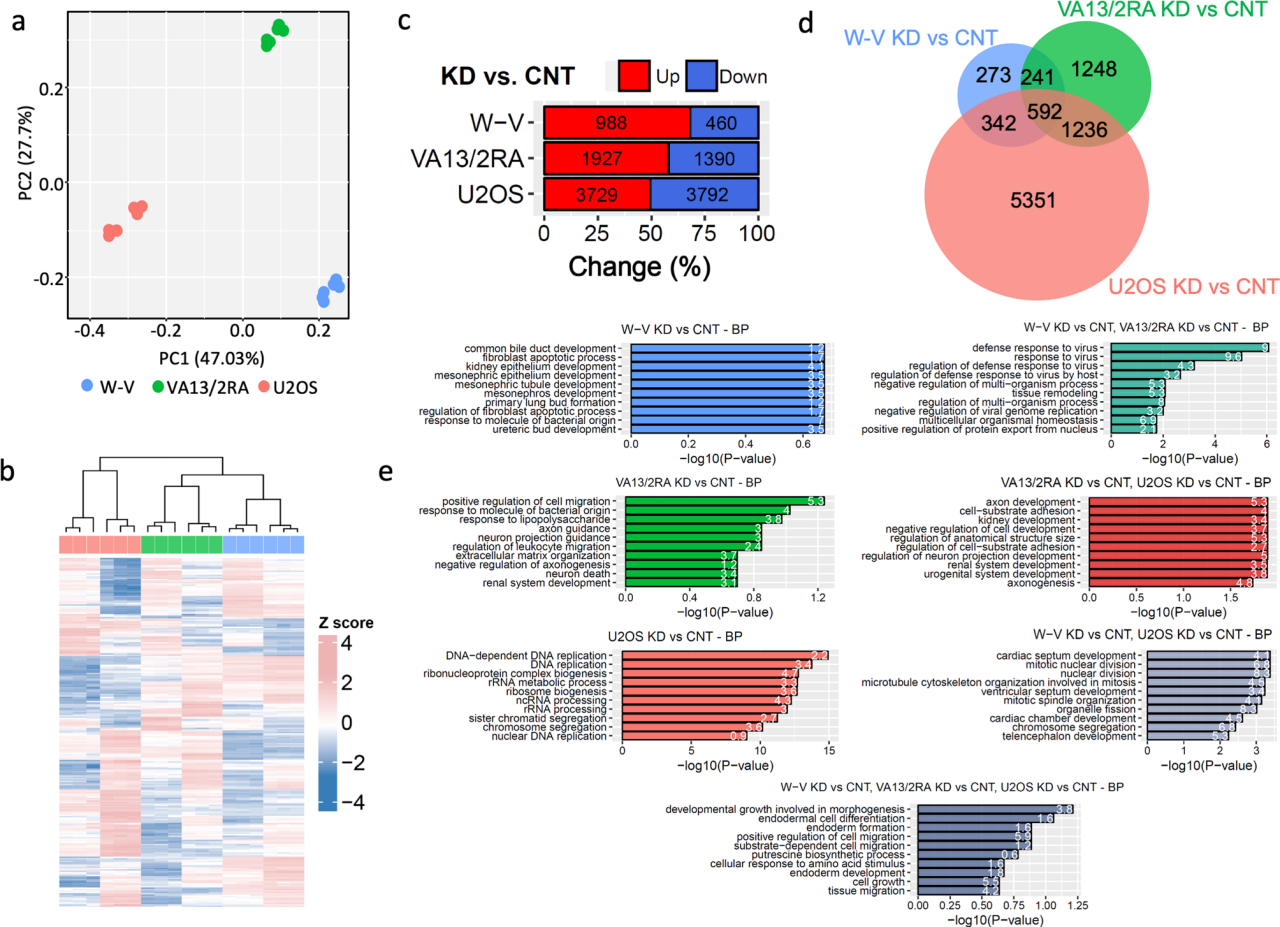
Overview of changes in gene expression upon NR2F2 downregulation in ALT+ cell lines. (**a**) Principal component analysis of the first two components, with WV (blue), VA13/2RA (green), and U2OS (red). (**b**) Hierarchal clustering heatmap of the top 100 differentially expressed genes between NR2F2-KD and CNT in each cell line, normalized as z-score per gene in each biological replicate. (**c**) Percentage and number of upregulated (red) and downregulated (blue) differentially expressed genes (absolute log2 FC > 0.5, FDR < 0.05) between NR2F2 siRNA (KD) vs Control siRNA (CNT) for each cell line. (**d**) Venn diagram showing differentially expressed genes shared or limited to one cell line (**e**) Graphs showing the top 10 processes identified by gene ontology (GO) enrichment analysis from the uniquely DE genes in the each cell line (shown in **d**), panels on left in order W-V (blue), VA13/2RA (green), U2OS (pink). Panels on the right show GO enrichment analysis for the DE genes shared between pairs of cell lines (shown in **d**), panels on right W-V&VA13/2RA (teal), VA13/2RA & U2OS (red), W-V& U2OS (grey) cell lines. Finally, the bottom central panel (grey-blue) shows the GO enrichment analysis for the DE genes shared between all three cell lines (shown in **d**). For each panel the x-axis shows the negative log_10_ transformation of the enrichment *P* value. The number on each bar shows the gene ratio (the percentage of DE genes associated with the GO term).

To reconcile the different responses of the three ALT+ cell lines to NR2F2 depletion, differential gene expression (DE) analysis was conducted between control and siNR2F2 treated cells and comparisons made between cell lines. Following checks on the RNA-seq data (Supplementary Figure S7 and Supplementary Figure S4e), principal component analysis revealed three distinct expression profiles among the ALT+ cell lines, irrespective of NR2F2 depletion. The first principal component, accounting for 47% of the variation, separated the expression profiles of the U2OS cells (control and NR2F2 depleted) from the W-V and WI38VA13/2RA cell lines (Fig. 4a). This is consistent with different origins of these cells lines as U2OS was derived from an osteosarcoma^50^, whereas W-V and VA13/2RA were both derived as SV40-LT transformed fibroblast cells^51,52^. The hierarchical clustering of the top 100 DE expressed genes from the NR2F2 knockdown (NR2F2-KD) versus control (CNT) separated the U2OS from the W-V and VA13/2RA cells, recapitulating the PCA analysis. There was also good separation between NR2F2-KD vs CNT replicates for each cell line (Fig. 4b).

DE analysis between U2OS NR2F2-KD and CNT cells (absolute log2 FC > 0.5, FDR < 0.05) showed that the highest number of DE genes (n = 7521;), with fewer DE genes identified between the VA13/2RA KD vs CNT cells (n = 3317) and even fewer between the W-V KD vs CNT cells (n = 1448; Fig. 4c,d). Gene Ontology (GO) enrichment analysis of the DE genes showed that different biological process were influenced by NR2F2 depletion in each cell line (Fig. 4e). GO term analysis of the 5351 DE genes unique to U2OS revealed the top 10 enriched processes included DNA-dependent DNA replication (82 genes, *P* = 1.46e−19), rRNA processing (117 genes, *P* = 9.90e−16), and sister chromatid segregation (106 gene, *P* = 5.83e−15) (Fig. 4e and Supplementary Figure S8). In contrast, the 273 DE genes following NR2F2 depletion in W-V cells were enriched for GO terms relating to kidney and mesonephros development and the 1248 DE genes in VA13/2RA were associated with terms relating to neuronal and cell migrations (Fig. 4d,e). These are all processes previously shown to be influenced by NR2F2 expression^47,53^.

In agreement with the origins of the cell lines, the 241 DE genes shared between W-V and VA13/2RA were enriched for GO terms involved in defence against viruses (*P* = 2.80e−10; Fig. 4d,e and Supplementary Figure S9). Whereas the 342 DE genes shared between W-V and U2OS were enrichment for GO terms linked to mitotic nuclear division (*P* = 1.47e−07; Fig. 4d,e, Supplementary Figure S9). However, two of the genes linked to mitotic nuclear division showed opposite DE changes: the Heat Shock Protein Family A (*HSPA1A*) [log2FC 0.99, FDR 3.32e-03] was upregulated in WV and downregulated U2OS; while the Bone Morphogenetic Protein 4 (*BMP4*) [log2FC 1.27, FDR 3.33e-07] a secreted ligand of the TGF-beta, was down regulated in WV but upregulated in U2OS (Supplementary Figure S9). This is consistent with minor perturbations of the W-V cell cycle compared to the near cycle arrest in U2OS cells upon downregulation of NR2F2. The 592 DE genes share by all three ALT+ cell lines did not show GO term enrichment for genes involved in DNA replication or repair (Fig. 4d,e).

To investigate the cell cycle effect further, gene pathway enrichment analysis (GSEA) was used to determine how NR2F2 depletion influences cell cycle regulation. This revealed down-regulation of 42 cell cycle related genes in the W-V KD vs CNT (FDR = 0.0177), 57 in the VA13/2RA KD vs CNT (FDR = 0.0096), and 122 in U2OS KD vs CNT (FDR = 0.0024) (Supplementary Figure S10). Among these, 16 genes with roles in the cell cycle were down-regulated in all three cell lines. Notably 79/122 down-regulated cell cycle-related genes were unique to U2OS and included genes involved in cell cycle checkpoints, initiation of DNA replication, kinetochore proteins and mitotic chromosome segregation (Supplementary Figure S10 and Supplementary Table S2).

### Investigating NR2F2 depletion and ALT-associated gene expression

**Figure 5:**
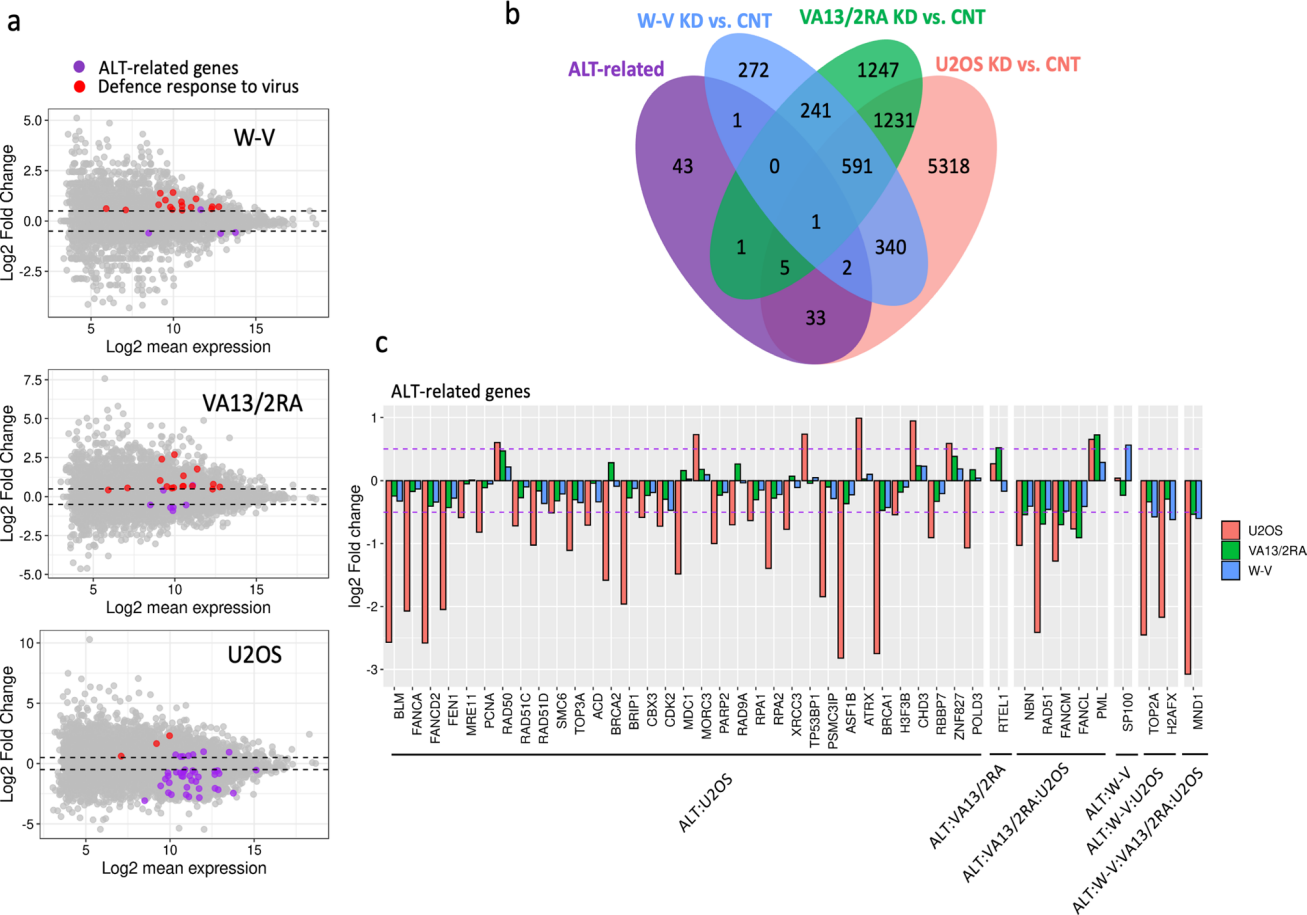
Identification of ALT-associated genes that show DE following NR2F2 depletion in each cell line. (**a**) MA plots of DE genes identified from the NR2F2-KD versus CNT comparison for each cell line. The ALT-related genes that showed significant DE are shown as purple dots and significantly DE genes involved in defence against viruses are shown as red dots. For each graph significantly DE genes appear above or below the dashed lines (absolute log2 fold-change > 0.5, FDR < 0.05). (**b**) Venn diagrams of the overlap between NR2F2-KD vs CNT differentially expressed genes (absolute log2 FC > 0.5, FDR < 0.05) with the list of 86 ALT-associated genes. (**c**) Bar-plot of significant changes in gene expression of ALT-associated genes. Dashed purple line represents the threshold of absolute log2 FC > 0.5.

The intersection of 9283 DE genes between NR2F2-KD and CNT in ALT+ cell lines with a list of 86 ALT-associated genes (Supplementary Table S3) identified 41 DE ALT-associated genes in U2OS but only seven in VA13/2RA and four in W-V (Fig. 5). Most (34/41) of the DE ALT-associated genes in U2OS were down-regulated with 10 genes showing a greater than 2 log2 fold change, including: *BLM*, *FANCA*, *FANCD2*, *FEN1*, *ASF1B*, *BRCA1*, *RAD51*, *TOP2A*, *H2AFX* and *MND1*. In contrast the *RAD50*, *MORC3, TP53BP1, CHD3, ZNF827* and *PML,* genes were all upregulated in U2OS NR2F2 depleted cells (Fig. 5c), as was ATRX but due to a large deletion this would not produce a functional protein^40^.

Five DE ALT-associated genes were shared by VA13/2RA and U2OS following NR2F2 depletion and just two by W-V and U2OS. Unexpectedly only one ALT-associated gene, *MND1,* showed consistent DE following NR2F2 depletion resulting in down-regulation in all three cell lines (Fig. 5b,c).

## Discussion

The two orphan nuclear receptors NR2F2 and NR2C2 have the potential to bind to telomeric DNA that contain (TCAGGG) variant repeats. The binding of NR2F2 and/or NR2C2 to telomeres in cells that utilise the ALT mechanism^6,30,32^ has been proposed to facilitate telomere-telomere interaction via recruitment of ZNF827 and the NuRD protein complex in the WI38VA13/2RA cell line^31^. Here, we found highly variable expression of NR2F2 but not NR2C2 among the five ALT+ cell lines investigated. The percentage of nuclei with detectable NR2F2 or NR2C2 foci was also highly variable between ALT+ cell lines (NR2F2 4.5–95%; NR2C2 10–52%), with the highest level in VA13/2RA, making it an outlier. There was no obvious relationship between the level of NR2F2 protein expression and the percentage of cells showing nuclear foci however, when present, the foci where significantly more likely to colocalise with telomeres in ALT+ cell lines compared to the telomerase + HT1080 cell line, although the VA13/2RA cell line was again an outlier. Consistent with the variability of those features, we also demonstrated that four of the five ALT+ cell lines have some telomeres that lack (TCAGGG) variant repeats making then unlikely sites for NR2F2 or NR2C2 binding. Following siRNA mediated depletion of NR2F2 there were some changes to the level ALT markers but the direction of change was inconsistent between cell lines. In brief, APB frequency increased in U2OS, decreased in VA13/2RA and was unchanged in W-V siNR2F2 depleted cells, whereas C-circle abundance increased in W-V and U2OS but was unchanged in VA13/2RA following NR2F2 downregulation. Western blot and differential gene expression analyses between siRNA control and siNR2F2 treated cells verified significant down-regulation of NR2F2 but no significant change in NR2C2 expression, indicating no compensatory effect in the cell lines examined.

The consequence of siNR2F2 treatment on gene expression varied considerably between the three ALT+ cell lines with the greatest perturbation in U2OS cells. PCA analysis of differential expression data following NR2F2 depletion showed a greater similarity between the VA13/2RA and W-V cell lines than to the U2OS. This is consistent with the GO term analysis showing enrichment for genes involved in defense against viruses, as both W-V and VA13/2RA cell lines are derived from SV40-LT transformed fibroblast cells. Depletion of NR2F2 in W-V and VA13/2RA affected the cell cycle to a limited extent compared to the effect in U2OS cells. This may be explained by SV40-LT expression, which disrupts the *TP53* and *RB1* signaling pathways^54^, giving rise to the W-V and VA13/2RA cell lines that have evolved independently to manage the disruption caused to the cell cycle. In contrast NR2F2 depletion had a significant effect on the cell cycle in U2OS cells, such that the U2OS cells were unable to enter S-phase, they accumulated in G1 or G2 consistent with the increase in APBs and C-circles. This conclusion is supported by the GO term analysis as the DE genes in the U2OS siNR2F2, which showed enrichment for biological processes linked to DNA replication, aspects of RNA processing and sister-chromatid segregation.

There was almost no overlap between genes that showed DE following NR2F2 depletion and ALT-related genes across the three cell lines. The one exception was *MND1*, which showed significant down-regulation in all three cell lines. The MND1-HOP1 heterodimer is required for long-range homology searching by RAD51-bound telomeres in ALT+ cells so facilitating strand invasion and break-induced replication (BIR) that lengthens telomeres^44^. Interestingly, RAD51 was significantly down-regulated in two of the NR2F2-depleted cell lines (U2OS and VA13/2RA), Fig. 5. In a different route to telomere lengthening in ALT+ cells, PCNA and POLD can be loaded onto damaged telomeres (for example telomeres with 3′ recessed ends) prior to BIR^43^ . Expression of the *PCNA* and *POLD* genes were significantly down-regulated in U2OS siNR2F2 treated cells but not in VA13/2RA and W-V. In the third proposed ALT mechanism, NR2F2 bound to telomeres recruits ZNF827 and the NuRD complex, preventing a DNA damage response and facilitating chromatin remodelling and telomere-telomere interactions^31^. Two components of the NuRD complex showed DE but in opposite directions (*CHD3* up-regulated and *RBBP7* down-regulated) and only in U2OS NR2F2 depleted cells. The *ZNF827* gene was also significantly up-regulated in U2OS treated cells, which is surprising as the zinc finger protein has been reported to interact with NR2F2 at telomeres^31^. Recently it has been shown that FANCD2 can form a complex with NR2F2 or NR2C2, independently from the Fanconi anaemia (FA) core complex or monoubiquitination of FANCD2. When the FANCD2-NR2F2/C2 complex localises to telomeres in ALT+ cell lines, the complex recruits the MUS81 endonuclease and stimulates DNA synthesis at telomeres suggesting a role in telomere maintenance in this setting^48^.

If the effect of NR2F2 depletion is to block or reduce entry into S-phase when that checkpoint is intact, this would be expected to have an indirect effect on ALT markers (APBs and C-circles), as seen in U2OS cells. Moreover, the lack of consistency in response to NR2F2 depletion among the ALT+ cell lines (with the exception of MND1 down-regulation) suggests that NR2F2 only plays an indirect role in ALT. Nevertheless, it is reasonable to ask why NR2F2 and NR2C2 bind to telomeres and recruit ZNF827 and FANCD2, especially as this may lead to insertion of telomeric DNA elsewhere in the genome and cause instability^32^. Recently it has been shown that ZBTB10, a zinc finger containing protein, preferentially binds to (TTGGGG) another variant repeat commonly found at the proximal ends of telomeres^1,14^. ZBTB10 binds to telomeres, particularly in the ALT+ U2OS cell line, where it facilitates interaction with the TRF2/RAP2 complex but inactivation of ZBTB10 had a limited effect on gene expression, and no clear effect on telomere length or ALT markers^55^. It was proposed that the (TTGGGG)–ZBTB10–TRF2/RAP1 interaction may play a role in DNA damage response at telomeres in normal cells^55^. We speculate that NR2F2 and NR2C2 could play a similar roles at very short telomeres that contain (TCAGGG) but lack (TTGGGG) repeats. Localisation of NR2F2 or NR2C2 at such telomeres might recruit FANCD2 or ZNF827 in a DNA damage response rather than a performing a specific role in the ALT mechanism.

In summary we have shown that NR2F2 depletion has very variable effects on differential gene expression, the cell cycle and ALT markers in U2OS versus WV and VA13/2RA cell lines. This points towards the known role of NR2F2 in regulation of the cell cycle but suggests only an indirect role on the ALT mechanism. Whether or not NR2F2/NR2C2 binding to (TCAGGG)_n_ repeats plays an important role at short dysfunctional telomeres in normal cells remains to be determined.

## Material and methods

### Cell lines and culture conditions

W-V is an SV40 transformed, ALT+ cell line derived from normal fibroblasts from a 45-year-old male with Werner’s syndrome (WRN−/−). W-V was a gift from Roger R. Reddel. The WI38VA13/2RA (VA13/2RA) ALT+ cell line, derived from normal female lung fibroblasts transformed by SV40 large T antigen, was obtained from the European Collection of Authenticated Cell Cultures (ECACC). SUSM-1 ALT+ cell line, derived from male liver fibroblasts immortalized by chemical (4NQO) transformation was a gift from Olivia M. Pereira-Smith. SAOS-2 and U2OS are both female osteosarcoma derived ALT+ cell lines obtained from Paolo Salomoni. HT1080, a telomerase-positive cell line derived from a male fibrosarcoma was obtained from ECACC.

W-V, U2OS and HT1080 were grown in DMEM medium (Gibco, UK) with 10% Fetal Bovine Serum (Gibco, UK); SAOS2 in RPMI 1640 (Gibco, UK) with 10% FBS and VA13/2RA in MEM medium (Gibco, UK) with 10% FBS and 1X Non-Essential Amino Acids (Gibco, UK). The cells were culture at 37 °C in 5% CO_2_ and ambient O_2_ and subcultured by standard methods. Dead cells were assessed using Trypan Blue stain (final concentration 0.2%, 3mins).

### Antibodies and western blot analysis

The primary antibodies used were: NR2C2/TR4 (PPH0107B-00) and NR2F2/COUP-TF2 (PPH7147-00, Perseus Proteomics), GAPDH (AM4300, Ambion, UK) and Anti IgG-Horseradish peroxidase (HRP)-conjugated secondary antibodies (NA934 and NA931, GE Healthcare, UK). Cell lysates (~ 1 × 10^6^ cells) were made in RIPA buffer, quantified using the Bradford assay and diluted to 1–3 mg/ml in Laemmli loading buffer. SDS-PAGE electrophoresis and western blots were prepared using standard procedures^56^. The X-ray films were converted to TIFF images and analysed using ImageJ v1.5e software, with normalisation^57^.

### Co-localisation of NRs with telomeres and APB detection

Cells on coverslips (70–90% confluency) were processed for antibody detection of NR2F2 or NR2C2 with visualization using fluorescence conjugated secondary antibodies (Anti IgG-Alexa488 ab150113, Abcam, UK). Telomeres were detected by in situ hybridization to Cy3–OO-(CCCTAA)_3_ PNA probe (PANAGENE) in PNA hybridization solution (70% deionized formamide, 0.25% blocking agent (PerkinElmer), 10 mM Tris–HCl, pH7.2)^6^. Coverslips were mounted with ProLong Gold containing 5 μg/ml of DAPI. APBs were detected by colocalisation of PML (PML primary antibody, sc-966, Santa Cruz Biotechnology, USA; secondary antibody Alexa488 ab150073, Abcam, UK) with telomeres^58^.

### Microscopy and image processing

For colocalization, cells were visualised using an Olympus FV1000 confocal microscope and intensity profile plot analysis. Nuclei at interphase were captured in 15–20 Z planes in 0.25 μm increments for the green (AlexaFluor488), red (Cy3-Tel-PNA) and blue (DAPI) channels. For quantitative analysis, slides were screened using the Olympus cell scanning system to capture images of 100–1000 s of nuclei in blue, green and red channels. Co-localisation analysis was conducted using ImageJ v1.5e software with the Java plugin tool by Pierre Bourdoncle (https://imagej.nih.gov/ij/plugins/colocalization.html).

### Single telomere length analysis (STELA)

STELA-PCR was conducted as reported previously^59^ with some minor modifications. Each STELA-PCR contained 250–1000 pg genomic DNA in 25 μl volume. The PCRs were cycled as follows: 25 cycles of 96 °C for 20 s, 63 °C (for flanking primers XpYp E2, 17p6, 12q-STELA) or 66.5 °C (for allele-specific primers XpYp-427G/415C) for 30 s, 68 °C for 10 min. Each reaction was split into three identical 0.8% LE agarose gels that were processed into Southern blots with variant-repeat detection by hybridization to ^32^P-dCTP labeled DNA probes that detect (TTAGGG)_n_, (TCAGGG)_n_, or (TGAGGG)_n_ repeats. Phosphor-image analysis was conducted using a Typhoon 9400 (GE Healthcare) and ImageQuant software. N.B. the 17p6 primer annealing site is polymorphic (presence/absence) in populations. Consequently, productive amplification with 17p6 in STELA reactions behaves as a Mendelian trait (NJR unpublished).

### C-circle analysis

The C-circle assay^26^ was conducted and C-circle abundance quantified by dot-blot hybridisation of native and denatured products to ^32^P-dCTP labelled telomere and Alu probes respectively. The signal from the telomeric probe hybridization was normalised to the Alu probe using the ImageQuant software (GE Healthcare).

### FACS analysis of the cell cycle

Cell cycle analysis was carried out by Propidium Iodide (PI) staining and FACS at 72 h after transfection with the siNR2F2 or control siRNAs. Approximately 1 × 10^6^ cells were harvested in 1 ml of 70% ice-cold ethanol. The cells in PBS were treated with RNase, stained with PI, and processed using a FACSCantoII cell analyser (BD Biosciences). The analysis was carried out using the FACSDiva software (BD Biosciences).

### Statistical analyses

Data from western blots, c-circle assays, co-localisation of NRs with telomeres, APBs, and cell cycle analyses were assessed using ANOVA, pairwise multiple comparison T tests (with Benjamini–Hochberg correction), or Pearson correlations using programs in R v3.5.1.

### Design and delivery of siRNAs using lipid system

Short sequence BLAST against the human transcriptome was used identify potential NR2F2 siRNA sequences that avoid targeting other genes. The target sequence in NR2F2 exon 2 shows no significant similarities (< 60%) with other genes and includes 5 mismatches with NR2F1 transcripts^60^. It is predicted to target five known splice variants of NR2F2 that are protein coding (Supplementary Figure S4). The sequence of the NR2F2 targeting RNA oligonucleotides were antisense: 5′-CCACUCGUACCUGUCCGGAUAUAUU-3′ and sense 3′-GGUGAGCAUGGACAGGCCUAUAUAA-5′. The Stealth siNR2F2 and control oligonucleotides were synthesised and annealed by Thermo Fisher Scientific (Stealth RNAi siRNA Negative Control Med GC, Cat# 12935-300).

For each biological replicate nine 10 cm petri-dishes (each containing three coverslips) were seeded with cells and cultured for 24 h to reach 40–50% confluence. Prior to transfection, the medium was replaced. Three plates were used as untreated controls, or transfected with the NR2F2 targeting or control Stealth siRNAs. Transfection of the double-stranded siRNAs (25 to 50 pmol) was carried out in low toxicity Lipofectamine RNAiMAX Transfection Reagent with Opti-MEM Medium (Thermo Fisher Scientific) following the manufacturer’s instructions. The siRNA/lipofectamine mix was added to growth medium in a 1:5 ratio. After 72 h, the coverslips were removed for APBs analysis. The rest of the cells were detached from the petri-dish by trypsinisation and harvested for western blot (0.5 × 10^6^), C-circle (1 × 10^6^), RNA-Seq (1.5 × 10^6^ ) and FACS (1 × 10^6^) analyses.

### Transcriptome analysis by RNA-Seq

The integrity and purity of extracted RNA samples was assessed using an Agilent 2100 Bioanalyser with a Nano-RNA 6000 Chip and by OD260/OD280 Nanodrop readings. Total RNAs (2.5 μg per sample) were sent to Novogene Corporation (Hong Kong) for library preparation using an Illumina TruSeq protocol, including mRNA poly-A selection, mRNA random fragmented, and first strand cDNA synthesis with random hexamers and reverse transcriptase. The cDNA libraries were sequenced using an Illumina-HiSeq 4000, generating > 12 GB data.

### Trimming, mapping and read counting of RNA-seq data

Each cDNA library generated > 30million paired-end (PE) reads. Fastq files were trimmed using skewer v. 0.2.2^61^ and the fastq files assessed before and after removing the adaptors using FASTQC v0.11.5 (https://www.bioinformatics.babraham.ac.uk/projects/fastqc/). Remaining reads were aligned to the human genome with TopHat2^62^ using the human genome assembly (Homo_sapiens.GRCh38.82) as reference. Only uniquely mapped reads were used in the downstream analysis (Supplementary Figure S3). The counts of reads per gene was conducted using Rsubread.v.1.26^63^ to Homo_sapiens.GRCh38.82 gene annotations. All bioinformatic analyses were preformed using the University of Leicester’s high-performance computer.

### Differential gene expression and pathway enrichment analysis

The resulting count matrix from Rsubread.v.1.26 was imported into R and, to filter out low expressed genes, only genes with at least 1 count per million (CPM) reads in a minimum of three samples were used for further analysis. The Fragments Per Kilobase of transcript per Million mapped reads (FPKM) method^64^ by countToFPKM v.1.0 was used to obtain the normalised gene expression. Differential expression (DE) between the groups were determined with DESeq2 v1.16.1^65^. GO analysis was performed on gene subsets identified by overlap analysis of DE genes (absolute log2FC >  = 0.5, FDR < 0.05) using R/Bioconductor cluster Profiler v3.10.1^66^ and the top 10 terms for each gene set was plotted. Pathway enrichment analysis, using Gene Set Enrichment Analysis (GSEA) with the Reactome database of biological pathways, was conducted using DOSE v.3.10.1: an R/Bioconductor package for Disease Ontology Semantic and Enrichment analysis^67^.

The RNA-seq data has been deposited at EMBL-EBI ArrayExpress: accession number E-MTAB-9599 available at: https://www.ebi.ac.uk/arrayexpress/.

## Supplementary information

Supplementary Information.
