# Supplementary material for: The absence of (TCAGGG)n repeats in some telomeres, combined with variable responses to NR2F2 depletion, suggest that this nuclear receptor plays an indirect role in the alternative lengthening of telomeres
Source: Sci Rep. 2020 Nov 26;10:20597. doi: 10.1038/s41598-020-77606-w (PMC7691514; doi:10.1038/s41598-020-77606-w)
Supplement: Supplementary file 1 — Supplementary Information. [file 41598_2020_77606_MOESM1_ESM.pdf]

The absence of $(TCAGGG)_n$ repeats in some telomeres, combined with variable responses to NR2F2 depletion, suggest that this nuclear receptor plays an indirect role in the Alternative Lengthening of Telomeres

Ahmed S. N. Alhendi and Nicola J. Royle *
Department of Genetics and Genome Biology, University of Leicester, LE1 3HE

Supplementary figures S1 –S10

Supplementary Tables S1 - S3

Uncropped Western replica blots for Figure 1a and Figure 3a

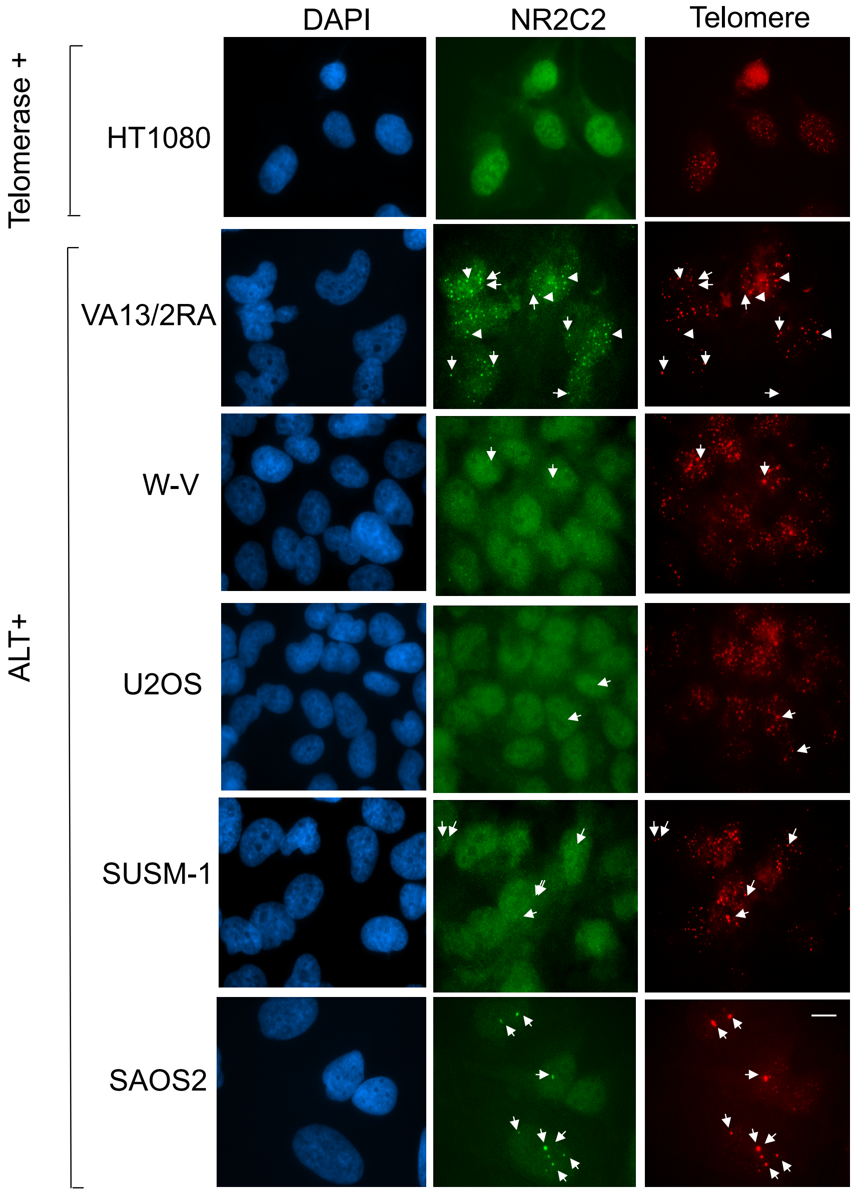

Supplementary Figure S1. Detection of NR2C2 foci and telomeres in six cell lines.

Immunofluorescence detection of NR2C2 foci (Alexa 488,green) and telomere foci by Telo-PNA hybridisation (Cy3–$(CCCTAA)_3$, red) in DAPI stained nuclei (blue) from the HT1080 (Tel+) and the VA132RA, W-V, U2OS SUSM-1 and SAOS2 (ALT+) cell lines. White arrows highlight possible sites of colocalisation. Scale bar 10mm.

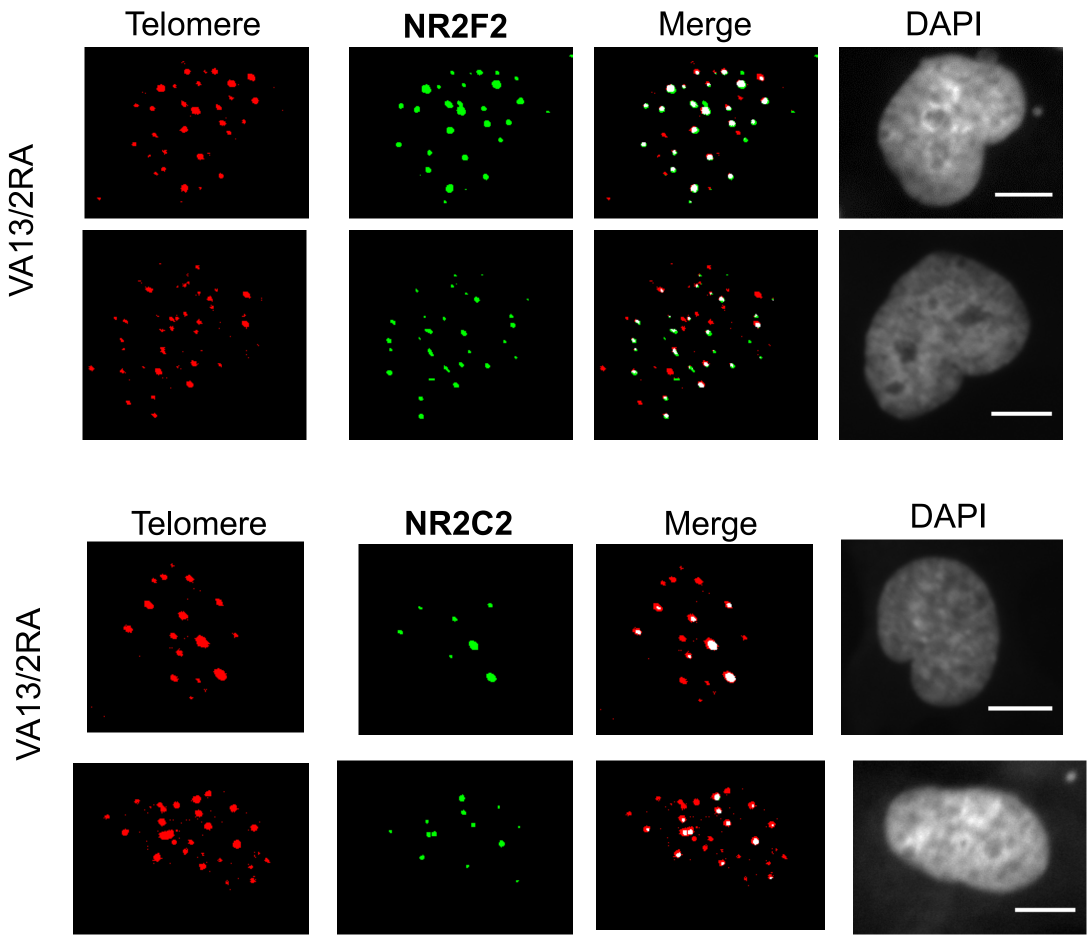

Supplementary Figure S2. Examples of NR2F2/NR2C2 –telomere colocalization analysis in VA13/2RA.

Slides were screened using the Olympus IX81 motorized microscope system with automated image acquisition software to capture images of 100s-1000s of nuclei in blue, green and red channels and analysed using ImageJ. Images shown are telomere foci detection by Telo-PNA hybridisation (Cy3–$(CCCTAA)_3$, red); NR2F2 or NR2C2 foci detection by immunofluorescence (Alexa 488,green). The merged images demonstrate colocalisation between some red and green foci (yellow). The DAPI stained nuclei are shown in panels on the right. Scale bar 10mm.

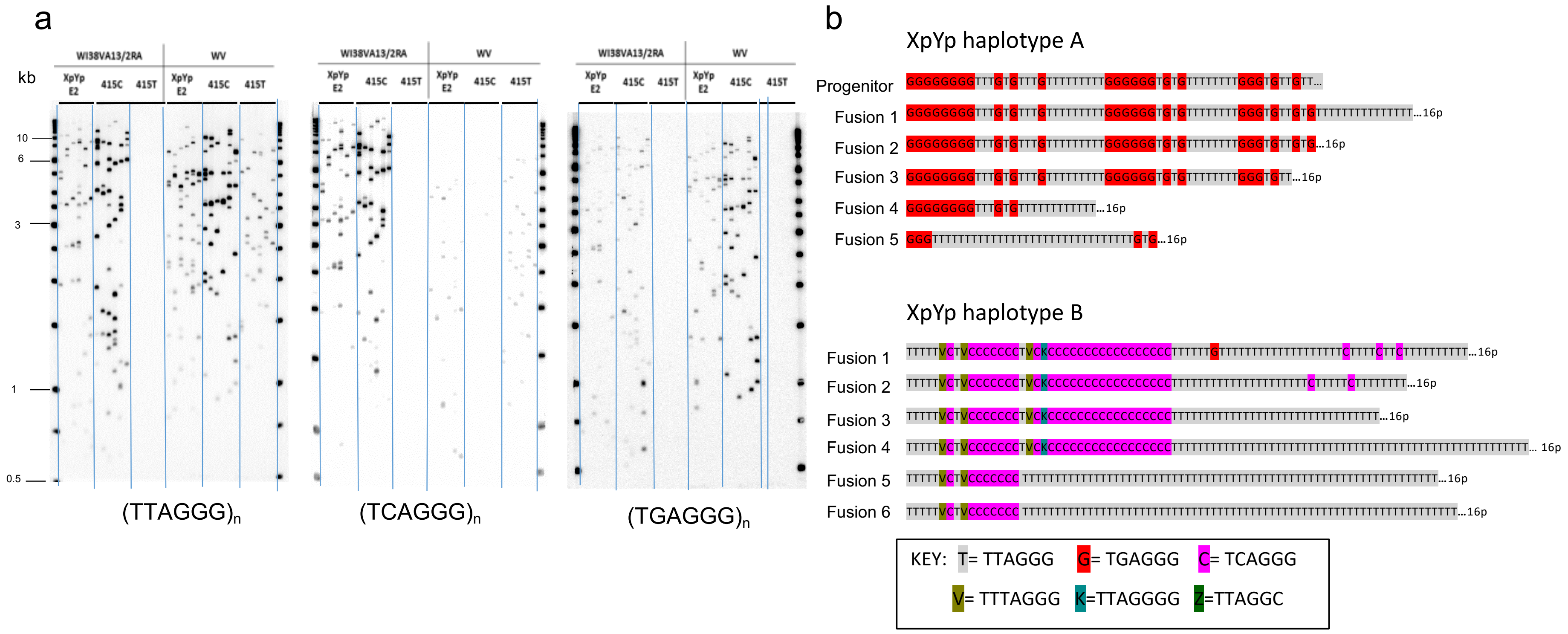

Supplementary Figure S3. XpYp telomere composition.

(a) Assessment of variant repeat composition in XpYp telomere alleles in theVA13/2RA and W-V cell lines. STELA was conducted using the chromosome specific (XpYp-E2) or the allele specific primers XpYp-427G/415C or XpYp-427A/415T. Southern blots of the amplified products were hybridised to different probes to detect $(TTAGGG)_n$, $(TCAGGG)_n$, and $(TGAGGG)_n$ repeats. VA13/2RA contains XpYp alleles that are amplified by the XpYp-427G/415C primer only, whereas both allele specific primers amplify telomere molecules in the W-V cell line. The W-V XpYp alleles amplified by the XpYp-427A/415T primer all contain $(TCAGGG)_n$ repeats whereas the molecules amplified by the XpYp-427G/415C allele specific primer do not.
(b) Verification of the allelic differences in variant repeat content of the W-V XpYp telomeres. Amplification and sequence analysis of the XpYp:16p telomere-fusion molecules from the W-V cell line showed that fusion molecules associated with the XpYp flanking haplotype B (-427A/-415T) included $(TCAGGG)_n$ repeats whereas fusion molecules associated with the flanking haplotype A (-417G/-415C) lack these repeats. Key shows the letter codes used for each repeat type e.g. T = (TTAGGG)

a

5'– CCACUCGUACCUGUCCGGAUAUAUU – 3' Anti-sense
3'– GGUGAGCAUGGACAGGCCUAUAUAA – 5' sense

b

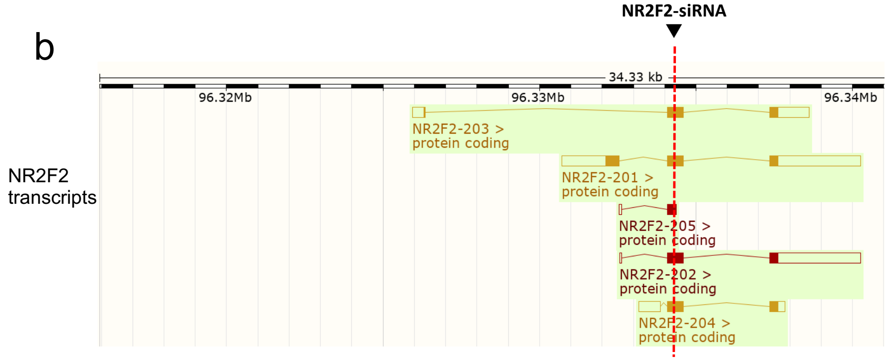

c

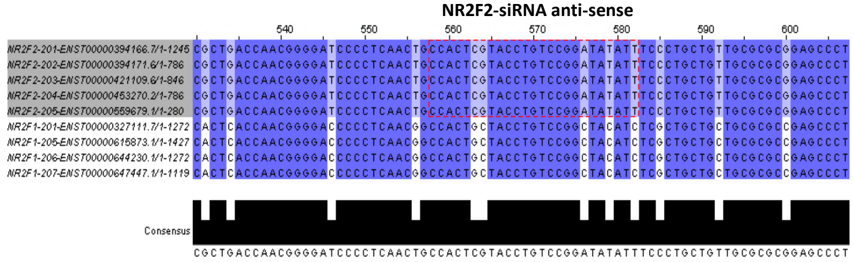

d

Description	Max Score	Total Score	Query Cover	E value	Per. Ident	Accession
Transcripts						
Homo sapiens nuclear receptor subfamily 2 group F member 2 (NR2F2), transcript variant 4, mRNA	50.1	50.1	100%	2e-05	100.00%	NM_001145157.1
Homo sapiens nuclear receptor subfamily 2 group F member 2 (NR2F2), transcript variant 3, mRNA	50.1	50.1	100%	2e-05	100.00%	NM_001145156.1
Homo sapiens nuclear receptor subfamily 2 group F member 2 (NR2F2), transcript variant 2, mRNA	50.1	50.1	100%	2e-05	100.00%	NM_001145155.1
Homo sapiens nuclear receptor subfamily 2 group F member 2 (NR2F2), transcript variant 1, mRNA	50.1	50.1	100%	2e-05	100.00%	NM_021005.3
PREDICTED: Homo sapiens potassium voltage-gated channel subfamily H member 7 (KCNH7), transcript variant X5, mRNA	26.3	26.3	52%	332	100.00%	XM_017005221.2
PREDICTED: Homo sapiens potassium voltage-gated channel subfamily H member 7 (KCNH7), transcript variant X4, mRNA	26.3	26.3	52%	332	100.00%	XM_017005220.2
PREDICTED: Homo sapiens potassium voltage-gated channel subfamily H member 7 (KCNH7), transcript variant X3, mRNA	26.3	26.3	52%	332	100.00%	XM_017005219.2
PREDICTED: Homo sapiens potassium voltage-gated channel subfamily H member 7 (KCNH7), transcript variant X2, mRNA	26.3	26.3	52%	332	100.00%	XM_017005218.2
PREDICTED: Homo sapiens potassium voltage-gated channel subfamily H member 7 (KCNH7), transcript variant X1, mRNA	26.3	26.3	52%	332	100.00%	XM_011512109.3

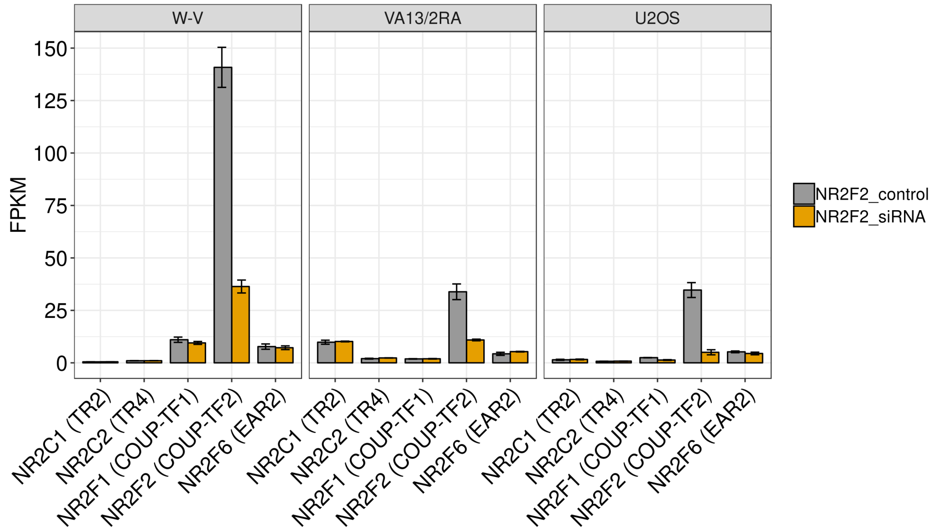

Supplementary Figure S4. Design of the NR2F2 stealth siRNA oligonucleotide sequence on NR2F2 and off-target assessment on human transcriptome and on related nuclear receptors.

(a) sequence of the stealth NR2F2 siRNA, (b) genomic location of the anti-sense NR2F2 siRNA target in relation to the five known alternative transcripts, which are protein-coding, (c) position of the anti-sense NR2F2 siRNA target on alignment of NR2F2 and NR2F1 cDNA sequences showing five nucleotide differences. Clustal-Omega v1.2.4 was used to generate the multiple sequence alignment of cDNA sequences, (d) NCBI short sequence BLAST of anti-sense NR2F2 siRNA target on the human transcriptome shows only significant matches with NR2F2 splice variants, (e) FPKM expression level of NR2F2 and other NRs upon NR2F2 downregulation in three identical experiments, Mean ± SD. There were no significant changes in gene expression of the other NR2F/C genes.

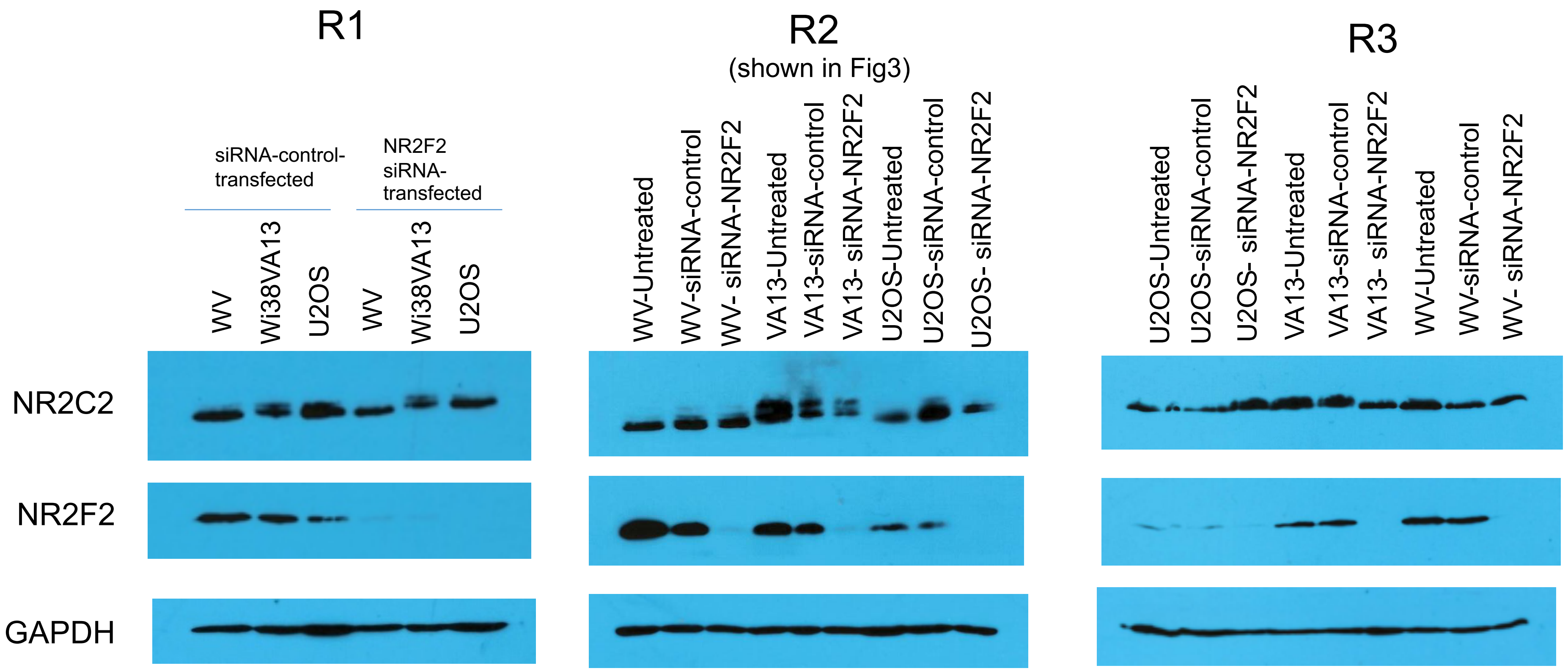

Supplementary Figure S5. Western blots of NR2C2 and NR2F2 in three replicates of siNR2F2 or siControl treated ALT+ cell lines.

Supplementary figure S6

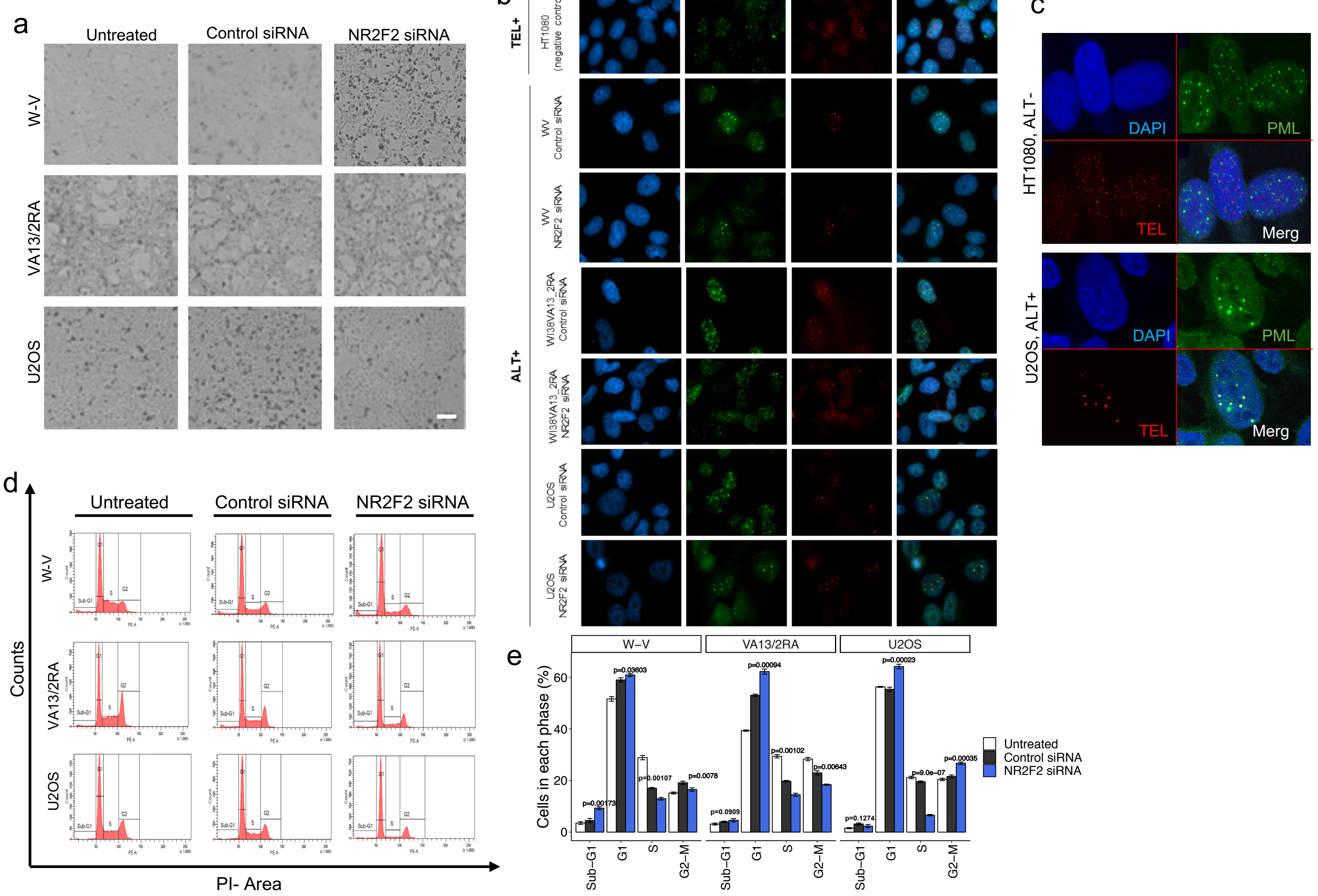

Supplementary Figure S6. Cell morphology, APB detection and FACS analyses of control and NR2F2 depleted ALT+ cell lines.
(a) Light microscopy images of untreated, control siRNA and siNR2F2 treated ALT+ cell lines after 72 hours, scale bar represents 100 μm.
(b) Detection of APBs upon downregulation of NR2F2 in ALT+ cell lines. Immunofluorescence staining of the PML protein (Alexa 488, green) and detection of telomeric DNA using a Telo-PNA probe (Cy3–$(CCCTAA)_3$, red) in interphase cells counterstained with DAPI (blue). Images captured using the Olympus IX81 motorised microscope system with automated image acquisition software.
(c) Example of confocal image analysis of APBs. A single slice from a confocal stack with resolution of 0.255 μm is shown. Blue channel (DAPI staining), green channel (immunofluorescence detection of PML with Alexa 488), red channel (telomere detection with Cy3-Telo-PNA).
(d) Cell cycle analysis of untreated, control siRNA and siNR2F2 treated cells from the three ALT+ cell lines. An example of the FACs density plots of cell cycle distribution after 72hr.
(e) Cell cycle analysis of untreated, control siRNA and siNR2F2 treated cells from the three ALT+ cell lines. n=3 independent experiments, error bars ± standard deviation. P values are from comparison of control siRNA vs siNR2F2 treated cells.

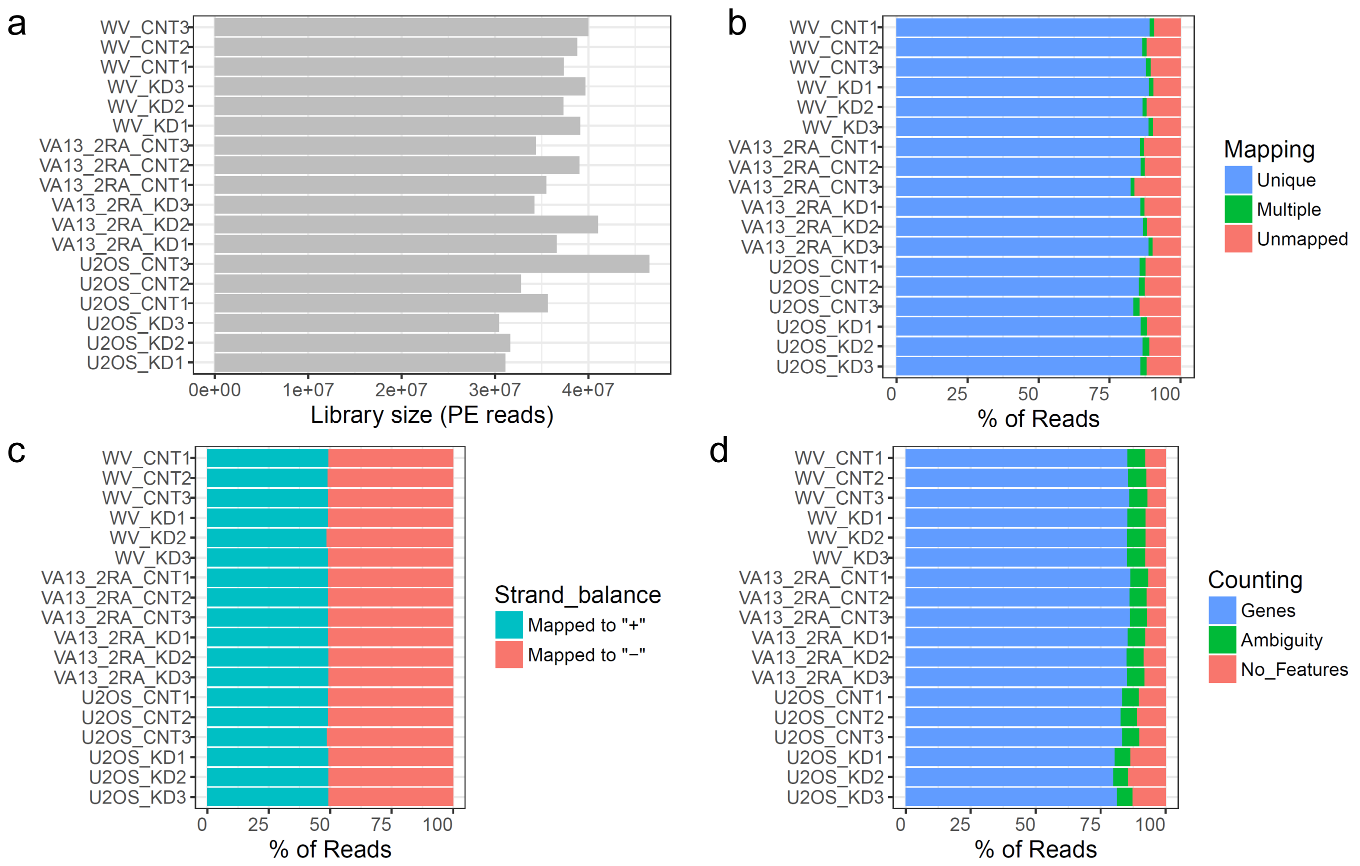

Supplementary Figure S7. Assessment of RNA-seq data quality from the triplicate control siRNA (CNT) and NR2F2 siRNA depleted (KD) assays for each ALT+ cell line.

(a) The sequencing library size represented by total number of PE (paired end) clean reads. (b) The mapping summaries for all PE clean reads. Unique represents the uniquely mapped PE sequences (before gene annotation); multiple is the fraction of PE reads that mapped to more than one site; unmapped represents the fraction of reads that were not mapped.
(c) The strand balance of mapped reads.
(d) Percentages of PE reads that mapped uniquely to genes. Overall, 83-87% were uniquely mapped to genes.

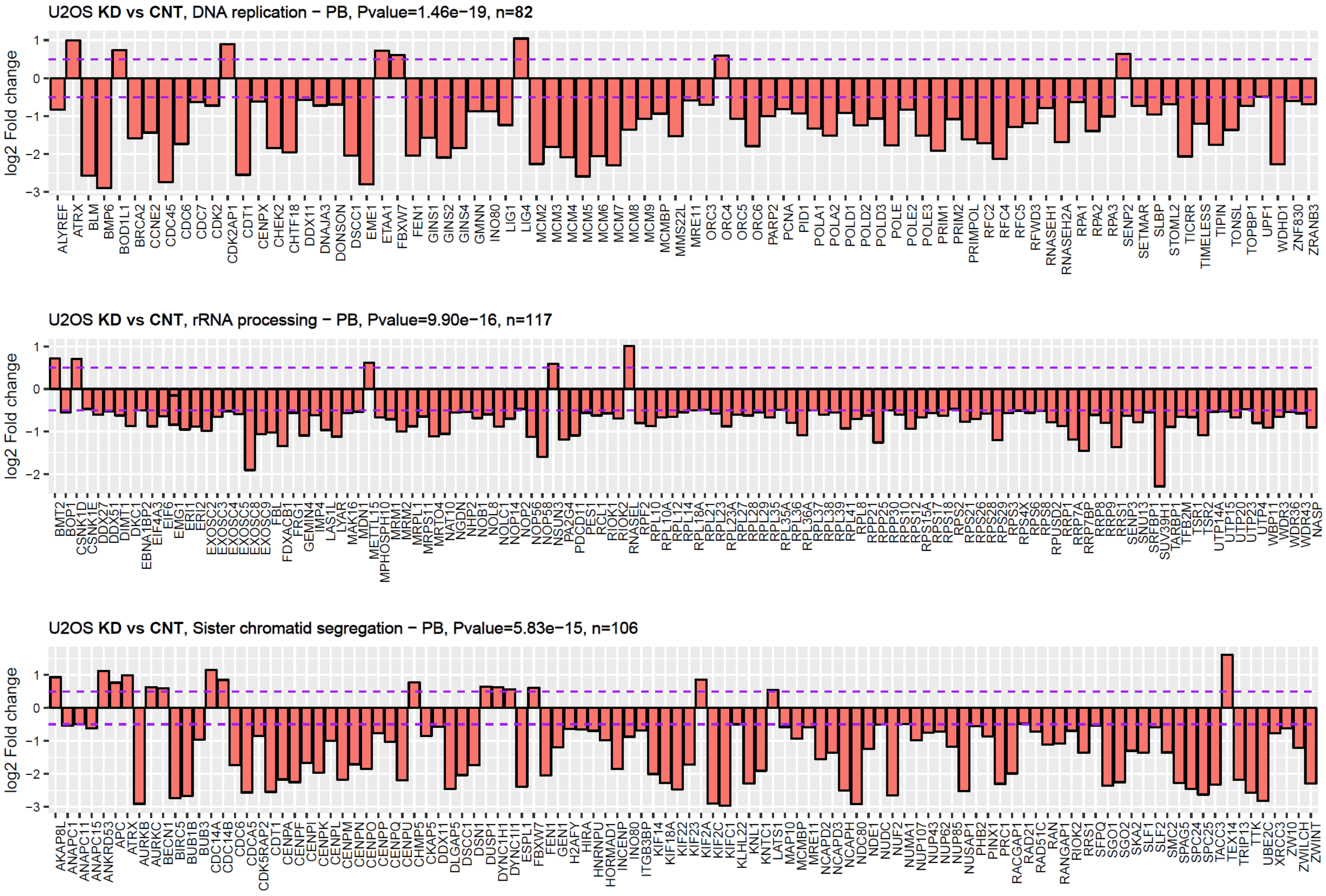

Supplementary Figure S8. Differentially expressed (DE) genes in U2OS that are associated with the top three enriched biological processes identified by GO analysis.

The bar chart shows the uniquely DE genes with a log2 fold change in level of gene expression between U2OS siNR2F2-KD and siRNA control (CNT). Dashed purple lines represent the threshold of absolute log2 FC > 0.5.

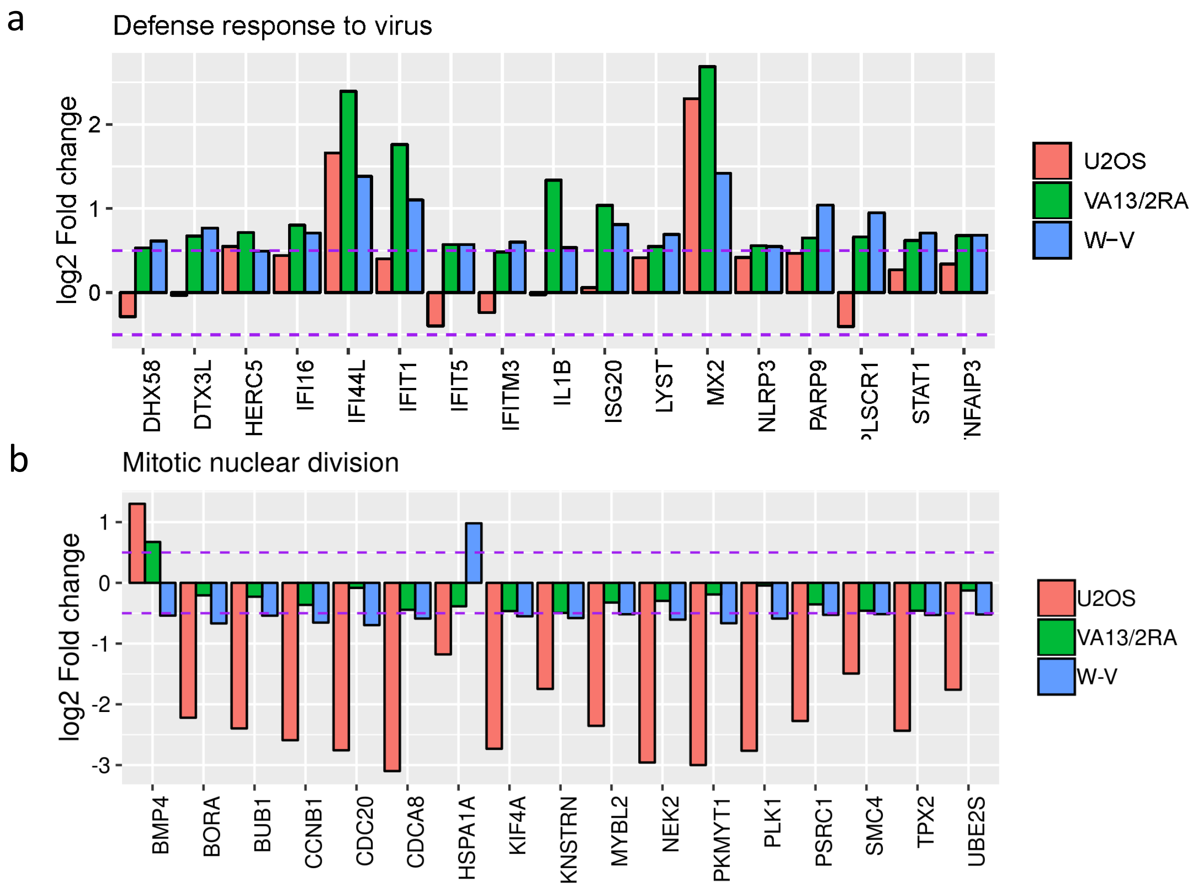

Supplementary Figure S9. Graphs showing the shared DE genes linked to the GO term defence against viruses in the VA13/2RA and W-V cell lines or to mitotic nuclear division in the W-V and U2OS cell lines.
(a) Expression changes in genes involved in defence against viral infections that are shared between the W-V KD vs CNT and VA13/2RA KD vs CNT. The change in expression of the same genes in U2OS are included for comparison.
(b) Expression changes in genes associated with mitotic nuclear division, detected in the W-V KD vs CNT and U2OS KD vs CNT comparisons. The change in expression of the same genes in VA13/2RA are included for comparison.
Both panels show comparison of the log2 fold change in expression between the siNR2F2 and siControl treated cells for each cell line. Dashed purple lines represent the threshold of absolute log2 FC > 0.5.

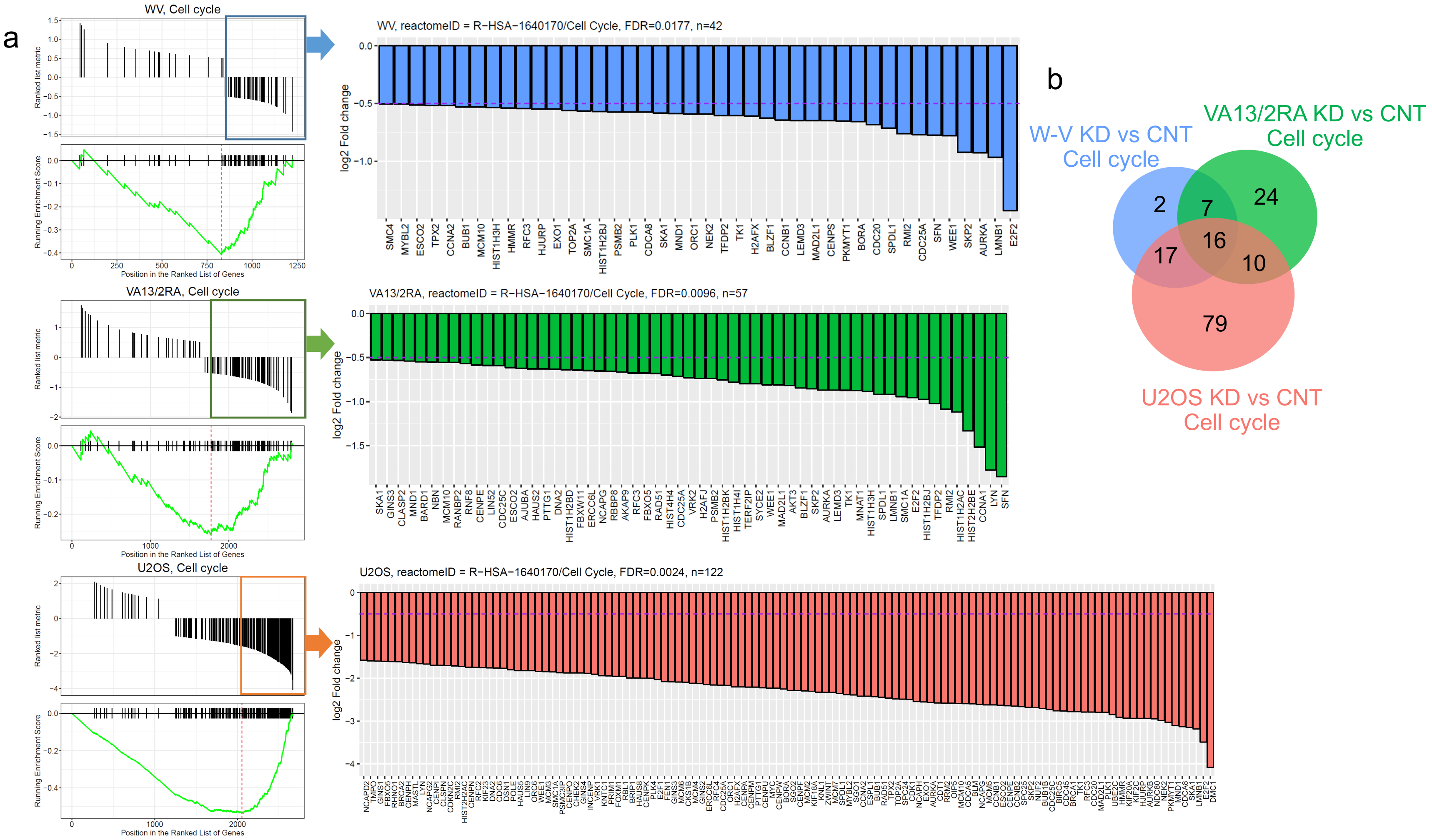

Supplementary Figure S10. Gene set enrichment analysis (GSEA) plots showing down regulation of the cell cycle, particularly in U2OS.
(a) on the right is the ranked metric of DE genes (top), and the curve (bottom) represents the enrichment score that reflects the degree to which a pathway is over represented in the ranked list of genes. Dashed red vertical lines shows the location of the maximum enrichment score. The coloured histograms (left) show the log2 fold gene expression change in leading-edge gene subsets. W-V-KD vs CNT (blue), VA13/2RA-KD vs CNT (green) and U2OS -KD vs CNT (orange).
(b) Venn diagram showing the overlap between the cell cycle genes identified by GSEA for each cell line, as shown in (a).

Supplementary Table S1. Distribution of NR2F/C2 foci at or outside telomeres

NR2C2 – Colocalisation

Cell line	Total no of analysed cells	Cells with NR2C2 foci (2+) %	Cells with NR2C2 colocalised foci with telomere (2-5) %	Cells with NR2C2 colocalised foci with telomere (6+) %	Cells with NR2C2 foci outside telomere (2-5) %	Cells with NR2C2 foci outside telomere (6+) %
**HT1080**	7229	4.22 ± 0.18	25.63 ± 1.10	3.25 ± 2.21	62.44 ± 6.55	13.98 ± 5.99
**W-V**	22081	9.82 ± 0.13	12.42 ± 1.12	86.16 ± 1.34	29.71 ± 1.29	28.75 ± 1.05
**VA13/2RA**	8264	52.11 ± 0.91	54.85 ± 3.42	8.94 ± 3.10	34.54 ± 1.03	6.05 ± 2.25
**U2OS**	21730	11.83 ± 0.40	72.25 ± 1.11	9.56 ± 1.11	19.63 ± 0.85	2.18 ± 0.76
**SUSM-1**	8715	12.16 ± 0.06	55.5 ± 2.17	12.62 ± 1.59	34.17 ± 1.68	3.76 ± 1.17
**SAOS2**	8809	15.77 ± 0.22	71.95 ± 4.97	5.01 ± 1.10	18.8 ± 2.01	2.85 ± 1.40

NR2F2 – Colocalisation

Cell line	Total no of analysed cells	Cells with NR2F2 foci (2+) %	Cells with NR2F2 colocalised foci with telomere (2-5) %	Cells with NR2F2 colocalised foci with telomere (6+) %	Cells with NR2F2 foci outside telomere (2-5) %	Cells with NR2F2 foci outside telomere (6+) %
**HT1080**	24765	2.43 ± 0.21	12.9 ± 1.73	1.85 ± 1.36	73.28 ± 6.96	16.96 ± 1.88
**W-V**	11904	16.18 ± 0.45	33.75 ± 2.4	7.61 ± 0.69	49.64 ± 1.07	7.63 ± 1.42
**VA13/2RA**	8512	94.33 ± 0.25	51.35 ± 1.34	36.14 ± 1.49	21.89 ± 0.54	3.78 ± 0.39
**U2OS**	32084	9.29 ± 0.25	46.33 ± 1.04	5.47 ± 0.38	45.07 ± 1.39	10.58 ± 0.64
**SUSM-1**	20655	17.73 ± 0.37	34.17 ± 1.68	3.76 ± 1.17	33.62 ± 1.41	5.24 ± 1.59
**SAOS2**	20473	4.48 ± 0.10	18.8 ± 2.01	2.85 ± 1.40	35.64 ± 4.81	17.39 ± 1.49

Total number of screened cells is from three independent experiments. Percentages represented by mean ± SD.

Supplementary table S2. Overlap of leading-edge differentially expressed genes associated with the cell cycle

Group	Number of DE genes	Gene symbol
U2OS:VA13_2RA:W-V	16	E2F2, AURKA, CDC25A, MND1, ESCO2, TK1, SKP2, LMNB1, RFC3, RMI2, SMC1A, SKA1, SPDL1, MCM10, WEE1, MAD2L1
VA13_2RA:W-V	7	SFN, TFDP2, HIST1H3H, PSMB2, LEMD3, HIST1H2BJ, BLZF1
U2OS:W-V	17	BORA, ORC1, CDC20, TPX2, CCNB1, TOP2A, H2AFX, HMMR, CCNA2, BUB1, EXO1, PKMYT1, MYBL2, NEK2, PLK1, HJURP, CDCA8
U2OS:VA13_2RA	10	RAD51A, CENPE, CDC25C, GINS3, LYN, NCAPG, PTTG1, ERCC6L, DNA2, FBXO5
W-V	2	CENPS, SMC4
VA13_2RA	24	HAUS2, HIST1H4I, CCNA1, NBN, HIST4H4, MNAT1, VRK2, H2AFJ, AKAP9, RBBP8, RANBP2, LIN52, HIST1H2AC, AJUBA, AKT3, TERF2IP, BARD1, CLASP2, HIST2H2BE, HIST1H2BK, FBXW11, SYCE2, HIST1H2BD, RNF8
U2OS	79	DMC1, MYC, ESPL1, TMPO, CDK1, CENPA, AURKB, NCAPH, CKS1B, MCM3, HAUS5, CDT1, MCM5, CENPW, CLSPN, CENPN, CENPH, RRM2, ZWINT, NDC80, SGO2, RHNO1, FEN1, SGO1, BLM, RFC2, PLK4, E2F1, BRCA2, RFC4, OIP5, HAUS8, NCAPG2, CDC45, PSMC3IP, PRIM1, NCAPD2, BRCA1, NUF2, GINS1, CDKN2C, CDC6, MCM6, SPC25, CHEK2, CENPI, KNL1, CENPU, MCM2, MCM4, VRK1, KIF18A, BRIP1, HIST2H2AC, MASTL, KIF2C, BIRC5, CENPM, KIF20A, CDCA5, GINS2, CENPO, RBL1, SPC24, CENPF, ORC6, INCENP, CENPK, UBE2C, LIN9, KNTC1, BUB1B, GINS4, FOXM1, CCNB2, KIF23, DSN1, MCM7, POLE

Supplementary Table S3. ALT-related genes

Gene Symbol	ENSMBL Gene ID	ALT requirement	Validation	Description	Reference
**BLM**	ENSG00000197299	YES	LKE	RecQ helicases	Bhattacharyya et al. 2009; Sobinoff et al 2017
**FANCA**	ENSG00000187741	YES	K	Fanconi Anaemia	Fan et al., 2009
**FANCD2**	ENSG00000144554	YES	K	Fanconi Anaemia	Fan et al., 2009
**FEN1**	ENSG00000168496	YES	K	Endonuclease	Saharia and Stewart, 2009
**MRE11A**	ENSG00000020922	YES	K	Subunit of MRN complex	Zhong et al., 2007, Henson et al., 2009
**MUS81**	ENSG00000172732	YES	K	Endonuclease	Zeng et al. 2009
**NBN**	ENSG00000104320	YES	K	NBS1 protein; Subunit of MRN complex	Zhong et al., 2007
**NSMCE2 / MMS21**	ENSG00000156831	YES	K	MMS21 Homolog; SMC5-SMC6 Complex	Potts & Yu, 2007
**PCNA**	ENSG00000132646	YES	K	Replication; RFC–PCNA functions as a telomere damage sensor	Jiang et al. 2009; Garcia-Exposito et al. 2016; Dilley et al. 2016
**RAD50**	ENSG00000113522	YES	LK	Subunit of MRN complex	Zhong et al., 2007, Henson et al., 2009
**RAD51A**	ENSG00000051180	YES	LK	RecA-like protein; a ss DNA-binding protein that promotes homology-directed searches and strand invasion at ALT telomeres	Draskovic et al. 2009; Cho et al., 2014
**RAD51C**	ENSG00000108384	YES	LK	RecA-like protein; a ss DNA-binding protein that promotes homology-directed searches and strand invasion at ALT telomeres	Draskovic et al. 2009; Cho et al., 2014
**RAD51D**	ENSG00000185379	YES	LK	RecA-like protein; a ss DNA-binding protein that promotes homology-directed searches and strand invasion at ALT telomeres	Draskovic et al. 2009; Cho et al., 2014
**SMC5**	ENSG00000198887	YES	K	SMC5-SMC6 Complex	Potts & Yu, 2007
**SMC6**	ENSG00000163029	YES	K	SMC5-SMC6 Complex	Potts & Yu, 2007
**TERF2**	ENSG00000132604	YES	LK E	TRF2 protein; Shelterin Complex Subunit ; ds telomere DNA binding; fusion; T-circles	Yeager et al 1999; Jiang et al. 2007; Stagno D'Alcontres et al. 2007
**TOP3A**	ENSG00000177302	YES	K	DNA Topoisomerase III Alpha	Raynard et al. 2006; Temime-Smaali et al. 2008
**SMARCAL1**	ENSG00000138375	YES	LK	ATP-dependent DNA-helicase	Poole et al. 2015; Cox et al. 2016

**POT1**	ENSG00000128513	-	L	Shelterin Complex Subunit	Denchi et al. 2007
**TERF1**	ENSG00000147601	-	LK	TRF1 protein; Shelterin Complex Subunit	Yeager et al 1999; Jiang et al. 2007
**TINF2**	ENSG00000092330	-	LK	TIN2 protein; Shelterin Complex Subunit,	Jiang et al. 2007; Temime-Smaali et al. 2008
**CTC1**	ENSG00000178971	-	K	Subunit of CTC1-STN1-TEN1 complex	Huang et al 2017
**STN1**	ENSG00000107960	-	K	Subunit of CTC1-STN1-TEN1 complex	Huang et al 2017
**TEN1**	ENSG00000257949	-	K	Subunit of CTC1-STN1-TEN1 complex	Huang et al 2017
**TOP2A**	ENSG00000131747	-	L	DNA Topoisomerase II Alpha	Bhattacharyya et al., 2009
**FANCM**	ENSG00000187790	-	LK	Fanconi Anemia	Xiaolei et al 2017
**ACD**	ENSG00000102977	-	L	TPP1 protein, Shelterin Complex Subunit	Hu et al. 2016
**ATM**	ENSG00000149311	-	L	Serine/Threonine Kinase	Stagno D'Alcontres et al. 2007
**ATR**	ENSG00000175054	-	L	Serine/Threonine Kinase	Barr et al. 2003
**BRCA2**	ENSG00000139618	-	L	DNA damage sensor	Wu et al. 2003; Acharya et al 2014
**BRIP1/ FANCJ**	ENSG00000136492	-	L	structure-specific DNA helicase	Déjardin et al. 2009
**CBX1/ HP1β**	ENSG00000108468	-	L	Heterochromatin Protein 1 Homolog Beta	Jiang et al. 2007
**CBX3/ HP1γ**	ENSG00000122565	-	L	Heterochromatin Protein 1 Homolog gamma	Jiang et al. 2007
**CBX5/ HP1α**	ENSG00000094916	-	L	Heterochromatin Protein 1 Homolog Alpha	Jiang et al. 2007
**CDK2**	ENSG00000123374	-	L	Cell cycle regulator	Wu 2003
**ERCC1**	ENSG00000012061	-	L	endonuclease; Excision Repair	Zhu et al 2003
**ERCC4/XPF**	ENSG00000175595	-	L	endonuclease; Excision Repair, T-circles	Sobinoff et al 2017
**FANCL**	ENSG00000115392	-	K	Fanconi Anaemia	Fan et al., 2009
**H2AFX**	ENSG00000188486	-	L	DNA damage response protein; TIFs	Nabetani et al 2004; Cesare et al 2009
**HUS1**	ENSG00000136273	-	L	Component of the 9-1-1 (RAD9-RAD1-HUS1) complex; checkpoint complex	Nabetani et al. 2004
**MDC1**	ENSG00000137337	-	L	Mediator of DNA Damage Checkpoint 1	Cesare et al 2009
**MORC3 /NXP2**	ENSG00000159256	-	L	MORC Family CW-Type Zinc Finger 3	Osterwald et al 2015
**PARP2**	ENSG00000129484	-	L	poly(ADP-Ribose) polymerase 2 (PARP-2); DSBR	Dantzer et al. 2004
**PML**	ENSG00000140464	-	L	Member of the tripartite motif (TRIM)	Yeager et al. 1999; Jiang et al. 2007

				family; TF; PML-nuclear bodies	
**RAD1**	ENSG00000113456	-	L	Subunit of RAD9-RAD1-HUS1 complex	Nabetani et al. 2004
**RAD17**	ENSG00000152942	-	L	clamp loader for the 9-1-1 (RAD9-RAD1-HUS1) complex	Nabetani et al. 2004
**RAD52**	ENSG00000002016	-	L	DNA binding protein; HR	Lundblad & Blackburn 1993; Yeager et al 1999; Min et al 2017
**RAD9A**	ENSG00000172613	-	L	Subunit of RAD9-RAD1-HUS1 complex	Nabetani et al. 2004
**RAD9B**	ENSG00000151164	-	L	Subunit of RAD9-RAD1-HUS1 complex	Nabetani et al. 2004
**TERF2IP /RAP1**	ENSG00000166848	-	L	Shelterin Complex Subunit ; TRF2-interacting protein aka RAP1	Jiang et al. 2007
**RIF1**	ENSG00000080345	-	L	RAP1 Interacting Factor Homolog; DDR for DSB	Silverman et al. 2004; Escribano-Díaz et al 2013
**RPA1**	ENSG00000132383	-	L	Subunit of the heterotrimeric RPA complex	Grudic et al. 2007
**RPA2**	ENSG00000117748	-	L	Subunit of the heterotrimeric RPA complex	Grudic et al. 2007
**TEP1**	ENSG00000129566	-	L	Telomerase-associated protein 1	Bhattacharyya et al., 2009
**WRN**	ENSG00000165392	-	LK	RecQ-like helicase and nuclease	Johnson et al. 2001; Mendez-Bermudez et al. 2012; Gocha et al. 2014
**XRCC3**	ENSG00000126215	-	K	Member of the RecA/Rad51-related protein family; T-circles	Compton et al. 2017
**RTEL1**	ENSG00000026036	-	LK	ATP-dependent DNA helicase implicated in telomere-length regulation	Vannier et al 2012
**PAXIP1**	ENSG00000157212	-	P	DNA damage response protein; **New ALT candidate**	Chu et al. 2017
**TP53BP1**	ENSG00000067369	-	LK	53BP1 protein; DNA damage response; TIFs	Nabetani et al 2004; Jiang et al. 2007; Cesare et al 2009
**PSMC3IP/HOP2**	ENSG00000131470	-	LK	Subunit of the PSMC3IP/MND1 complex	Cho et al., 2014
**MND1**	ENSG00000121211	-	LK	Subunit of the PSMC3IP/MND1 complex	Cho et al., 2014
**HSP90AA1**	ENSG00000080824	-	L	Heat Shock Protein HSP 90-Alpha	Bhattacharyya et al. 2009
**HSP90AB1**	ENSG00000096384	-	L	Heat Shock Protein HSP 90-Beta	Bhattacharyya et al. 2009
**ASF1A**	ENSG00000111875	-	LK	Anti-Silencing Function 1A Histone Chaperone	O'Sullivan et al. 2014
**ASF1B**	ENSG00000105011	-	LK	Anti-Silencing Function 1B Histone Chaperone	O'Sullivan et al. 2014

**ATRX**	ENSG00000085224	-	LK	Chromatin Remodeler	Lovejoy et al. 2012
**BRCA1**	ENSG00000012048	-	L	DNA damage sensor protein	Wu et al. 2003; Kargaran et al 2016
**DAXX**	ENSG00000204209	-	LK	Multifunctional protein; Transcriptional factor; ATRX partner	Lovejoy et al. 2012
**H3F3A**	ENSG00000163041	-	S	Histone variant H3.3a	Lovejoy et al. 2012
**H3F3B**	ENSG00000132475	-	-	Histone variant H3.3b	Lovejoy et al. 2012
**SP100**	ENSG00000067066	-	LKE	Major component of the PML bodies	Jiang et al. 2005
**TP53**	ENSG00000141510	-	LE	Tumour suppression; Transcription factor	Chen et al. 2006; Stagno D'Alcontres et al. 2007
**SLX4**	ENSG00000188827	-	LKE	Structure-Specific Endonuclease Subunit; T-circles	Vannier et al 2012; Sobinoff et al 2017
**HDAC1**	ENSG00000116478	-	-	NuRD complex	Conomos et al. 2014
**RBBP4**	ENSG00000162521	-	L	NuRD complex	Conomos et al. 2014
**MTA3**	ENSG00000057935	-	L	NuRD complex	Conomos et al. 2014
**HDAC2**	ENSG00000196591	-	L	NuRD complex	Conomos et al. 2014
**CHD3**	ENSG00000170004	-	-	NuRD complex	Conomos et al. 2014
**RBBP7**	ENSG00000102054	-	L	NuRD complex	Conomos et al. 2014
**MTA2**	ENSG00000149480	-	L	NuRD complex	Conomos et al. 2014
**CHD4**	ENSG00000111642	-	L	NuRD complex	Conomos et al. 2014
**MTA1**	ENSG00000182979	-	-	NuRD complex	Conomos et al. 2014
**MBD2**	ENSG00000134046	-	-	NuRD complex	Conomos et al. 2014
**MBD3**	ENSG00000071655	-	L	NuRD complex	Conomos et al. 2014
**ZNF827**	ENSG00000151612	-	LE	Zinc finger protein associated NuRD complex	Conomos et al. 2014
**POLD3**	ENSG00000077514	-	K	DNA Polymerase Delta 3, Accessory Subunit; PolD3 are required for BIR in ALT+ cell lines	Costantino et al., 2014

Total number = 86 genes. Validation Key: L= localization at telomeres in ALT cells; K= gene knockdown; E= gene expression; P= proteomics analysis; S= sequencing.ds

Figure 1a – uncropped western blots

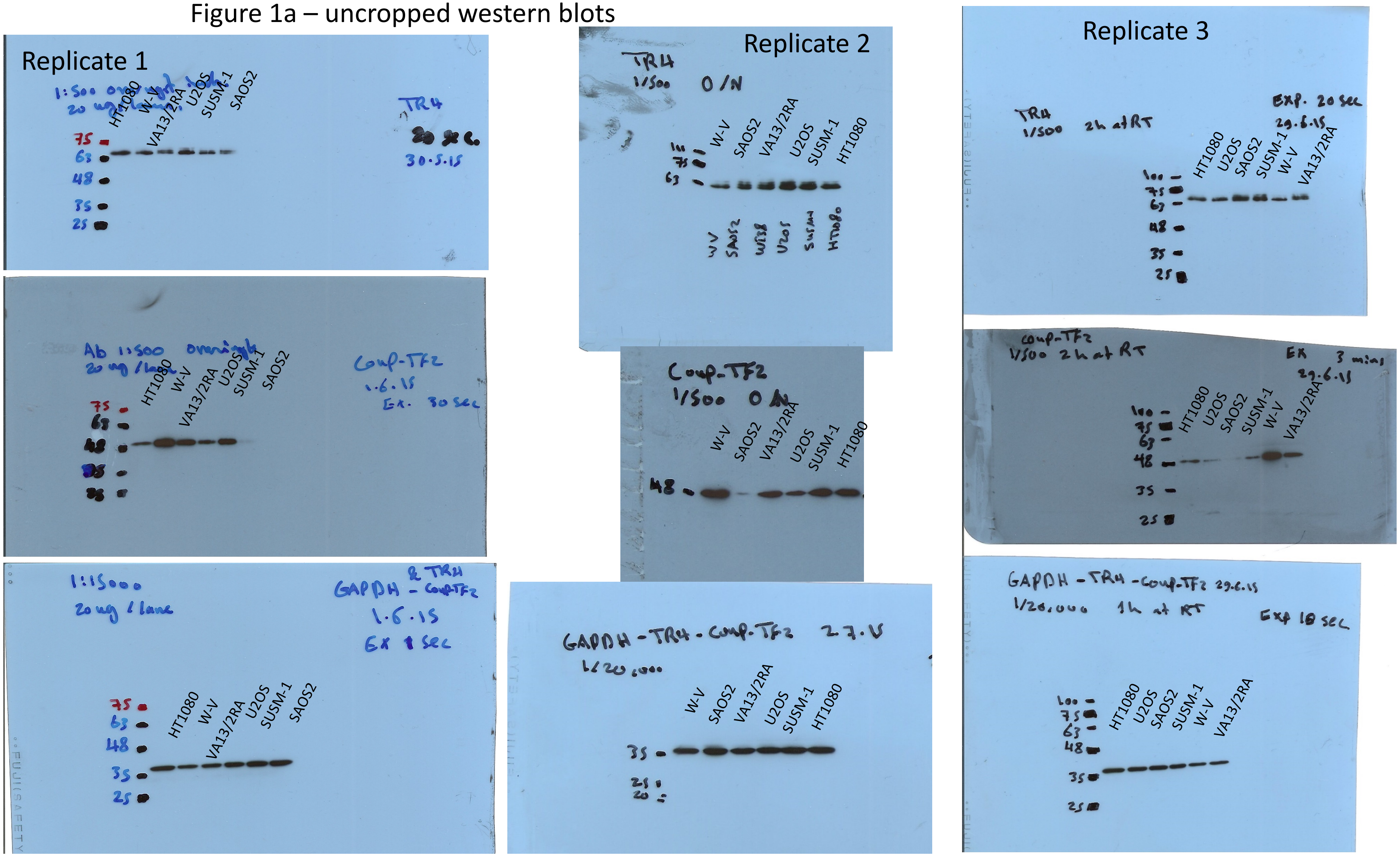

Figure 3a- uncropped western blots of NR2F2 & NR2C2 in siNR2F2 treated ALT+ cell lines

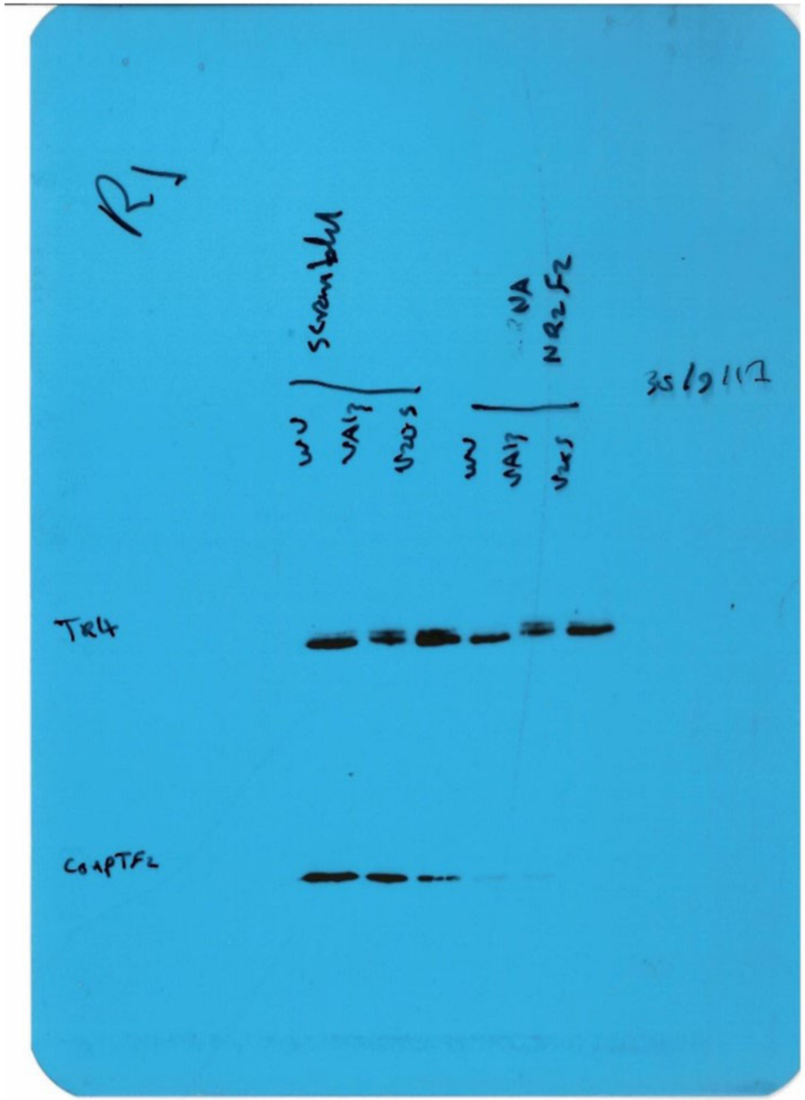

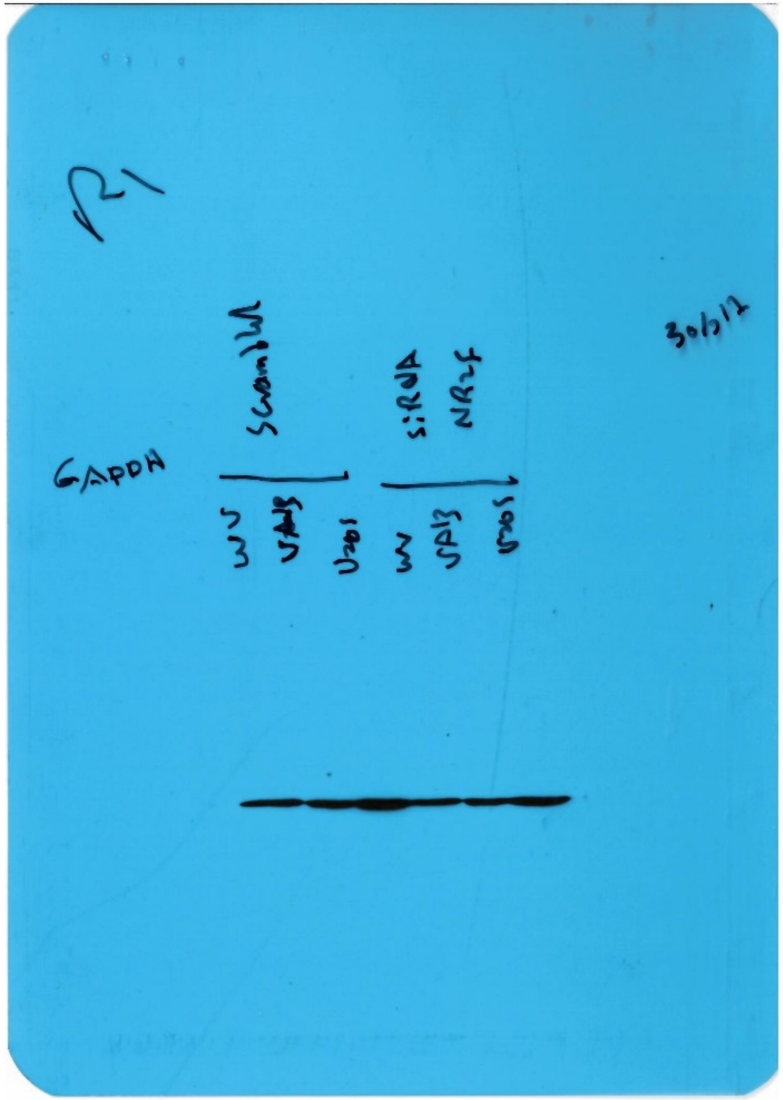

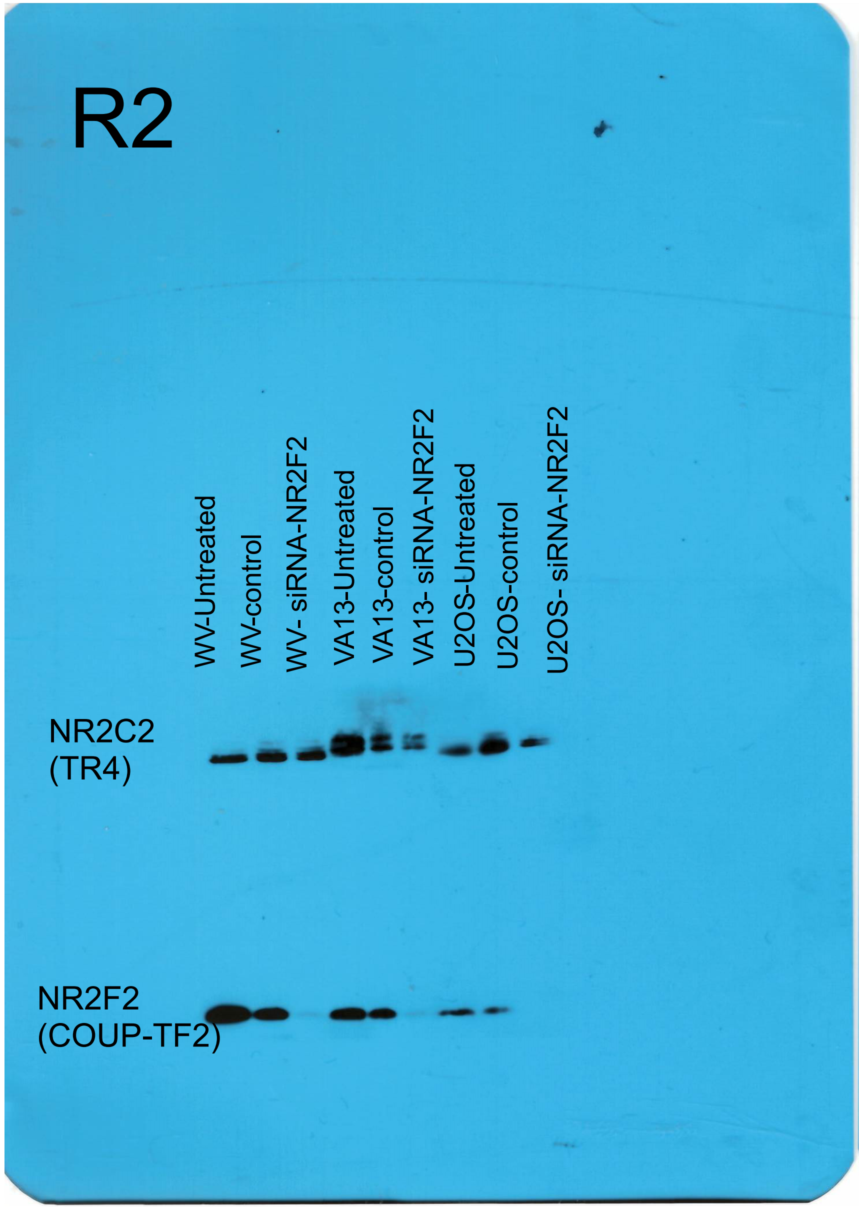
R2
WV-Untreated
WV-control
WV- siRNA-NR2F2
VA13-Untreated
VA13-control
VA13- siRNA-NR2F2
U2OS-Untreated
U2OS-control
U2OS- siRNA-NR2F2
NR2C2
(TR4)
NR2F2
(COUP-TF2)

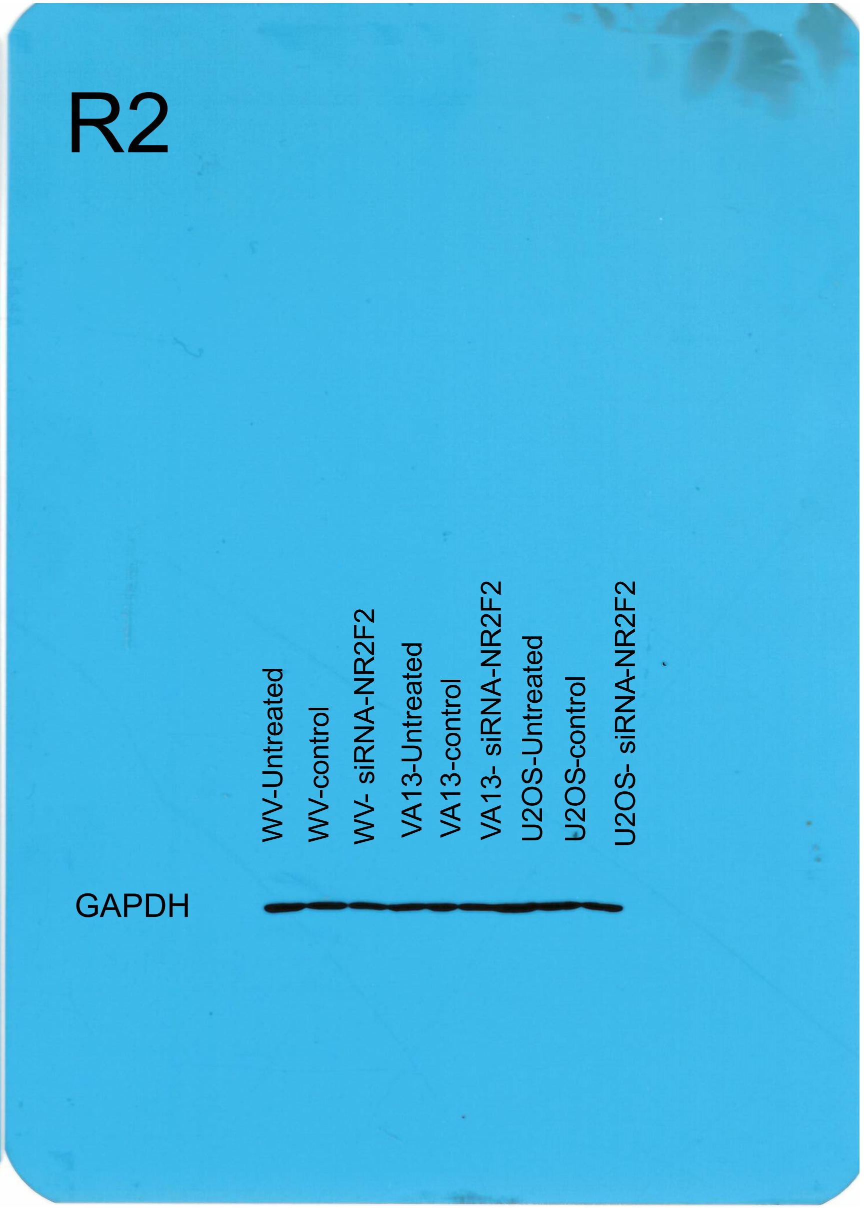
R2
WV-Untreated
WV-control
WV- siRNA-NR2F2
VA13-Untreated
VA13-control
VA13- siRNA-NR2F2
U2OS-Untreated
U2OS-control
U2OS- siRNA-NR2F2
GAPDH

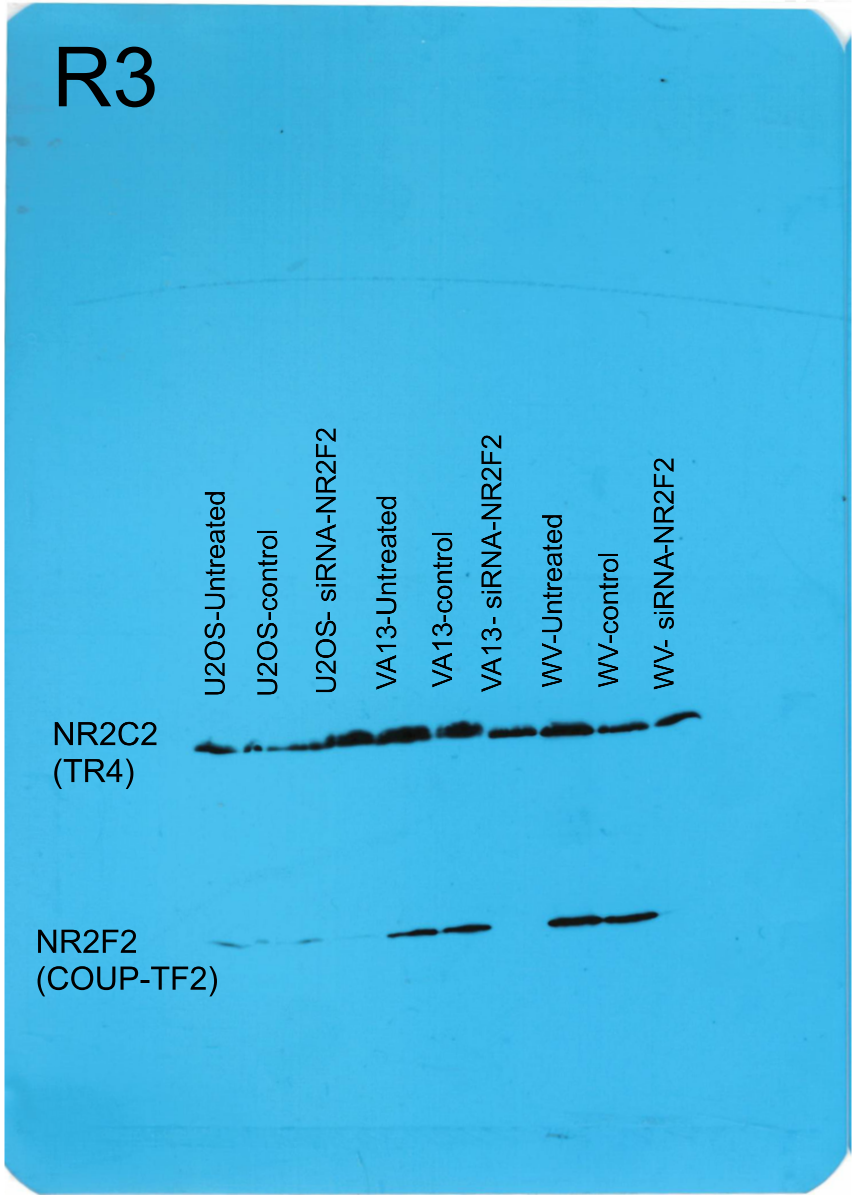
R3
U2OS-Untreated
U2OS-control
U2OS- siRNA-NR2F2
VA13-Untreated
VA13-control
VA13- siRNA-NR2F2
WV-Untreated
WV-control
WV- siRNA-NR2F2
NR2C2
(TR4)
NR2F2
(COUP-TF2)

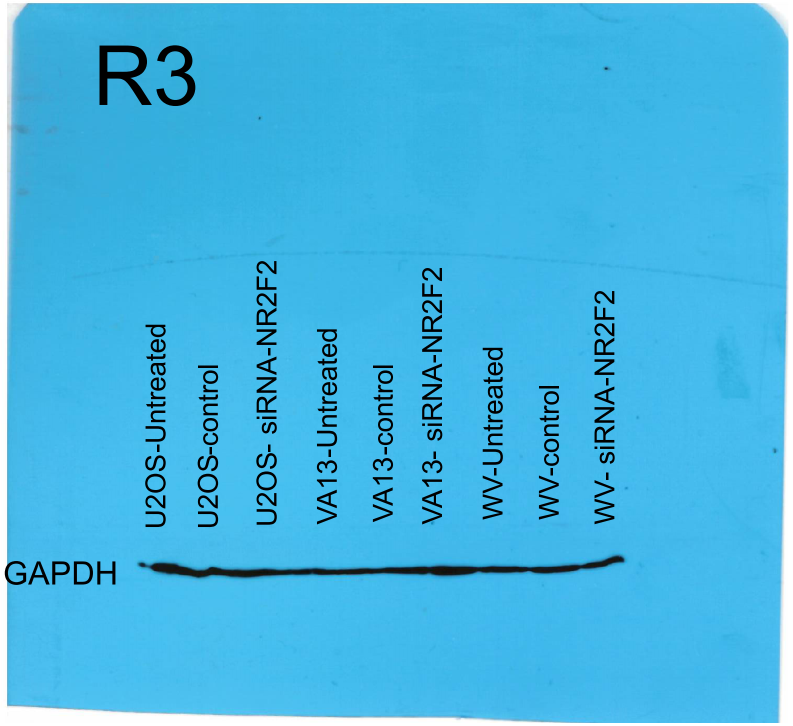
R3
U2OS-Untreated
U2OS-control
U2OS- siRNA-NR2F2
VA13-Untreated
VA13-control
VA13- siRNA-NR2F2
WV-Untreated
WV-control
WV- siRNA-NR2F2
GAPDH
